# SM-Omics is an automated platform for high-throughput spatial multi-omics

**DOI:** 10.1038/s41467-022-28445-y

**Published:** 2022-02-10

**Authors:** S. Vickovic, B. Lötstedt, J. Klughammer, S. Mages, Å Segerstolpe, O. Rozenblatt-Rosen, A. Regev

**Affiliations:** 1 Klarman Cell Observatory Broad Institute of MIT and Harvard, Cambridge, MA USA; 2 Department of Biology, Massachusetts Institute of Technology, Cambridge, MA USA; 3 New York Genome Center, New York, NY USA; 4 Science for Life Laboratory, Department of Biochemistry and Biophysics, Stockholm University, Solna, Sweden; 5 Science for Life Laboratory, Department of Gene Technology, KTH Royal Institute of Technology, Stockholm, Sweden; 6 Department of Biological Engineering, Massachusetts Institute of Technology, Cambridge, MA USA; 7 Howard Hughes Medical Institute and Koch Institute for Integrative Cancer Research, Department of Biology, Massachusetts Institute of Technology, Cambridge, MA USA; 8 Present Address: Genentech, 1 DNA Way, South San Francisco, CA USA

**Keywords:** Proteomics, Gene expression, Transcriptomics

## Abstract

The spatial organization of cells and molecules plays a key role in tissue function in homeostasis and disease. Spatial transcriptomics has recently emerged as a key technique to capture and positionally barcode RNAs directly in tissues. Here, we advance the application of spatial transcriptomics at scale, by presenting Spatial Multi-Omics (SM-Omics) as a fully automated, high-throughput all-sequencing based platform for combined and spatially resolved transcriptomics and antibody-based protein measurements. SM-Omics uses DNA-barcoded antibodies, immunofluorescence or a combination thereof, to scale and combine spatial transcriptomics and spatial antibody-based multiplex protein detection. SM-Omics allows processing of up to 64 in situ spatial reactions or up to 96 sequencing-ready libraries, of high complexity, in a ~2 days process. We demonstrate SM-Omics in the mouse brain, spleen and colorectal cancer model, showing its broad utility as a high-throughput platform for spatial multi-omics.

## Abstract (web summary)

The spatial organisation of cells and molecules plays a key role in tissue function. Here the authors report Spatial MultiOmics (SM-Omics) as a fully automated, high-throughput all-sequencing based platform for combined and spatially resolved transcriptomics and antibody-based protein measurements.

## Introduction

The spatial organization of cells and molecules is fundamental to physiological function and disease pathology, and imaging the position and level of molecules is a cornerstone of both basic biology and clinical pathology. Because gene expression is regulated at multiple levels from transcription to protein degradation, protein and RNA levels convey distinct information on gene function and cell state, as has been shown in diverse contexts including dynamic responses^1,2^, in genetic variation^3^, in human malignancies^4^, and in single cells in suspension^5^. Single cell genomics and multi-omics approaches, such as single cell and single nucleus RNA-Seq^6–11^ and CITE-Seq^5,12^, have been tremendously successful at capturing diverse molecular profiles at the level of individual cells and nuclei, but typically do not preserve spatial information. The importance of studying cells in their native environment has been shown in many processes, from normal organ development to spatial deregulation in diseases and often highlighted in the context of cancer propagation and resistance to therapy^13,14^.

Recent progress in spatial in situ profiling methods has opened the way for comprehensive profiling of location and expression simultaneously^15–28^. For spatial RNA measurements, Spatial Transcriptomics (ST)^24,26^ has emerged as a versatile approach for spatial RNA profiling. In ST, a fresh-frozen tissue section is placed on top of barcoded DNA primers attached to a glass surface^24^. Following tissue staining and histological imaging, cells are permeabilized, mRNAs are spatially tagged directly in tissues and a cDNA sequencing library is generated. After sequencing, the RNA-Seq information is traced back to the spatially barcoded positions on the glass slide providing a global spatial tissue profile. ST has been applied to diverse systems and tissue types, such as brain, heart, spinal cord, melanomas, breast cancer and prostate cancer^24,29–36^. However, barriers around throughput, resolution, and efficiency^37^, limit its application at large scale. In parallel, there have been advances in multiplex protein measurements in situ based on reading out multiple fluorescent-, heavy metal- or barcode coupled antibody tags^19,20,38–41^. Some methods rely on cyclic immunostaining or in situ sequencing barcoding schemes, whereas others use expensive machinery for Multiplexed Ion Beam Imaging or Imaging Mass Cytometry. Few platforms have combined RNA and antibody-based measurements to date^42–44^ and have traditionally relied on imaging one or the other modality. Companion technologies similar to our approach (e.g. Visium, 10X Genomics) rely on: (i) an antibody-based immunofluorescence (IF) read-out of 1–2 target antigens; (ii) do not employ DNA-barcoding strategies which allow us to parallelize antibody-based measurements, (iii) and process spatial RNA-Seq libraries manually, making these approaches low-throughput, laborious and not scalable due to intrinsic limitations of multiplex imaging.

To bridge this gap and make molecular tissue profiling a widely available and robust tool, we develop Spatial Multi-Omics (SM-Omics), an end-to-end framework that uses a liquid handling platform for high-throughput combined transcriptome and antibody-based spatial tissue profiling with minimum user input and available laboratory instrumentation^45,46^. SM-Omics relies on using DNA-barcoded antibodies, similarly to how CITE-seq^5^ performs simultaneous epitope and transcriptome profiling in single cells, to scale and combine spatial transcriptomics and spatial antibody-based multiplex protein detection. This user-friendly all-sequencing based technology allows processing of up to 64 in situ spatial reactions and up to 96 sequencing-ready libraries, of high complexity, in ~2 days, in a high-throughput platform for spatial multi-omics.

## Results

**Fig. 1:**
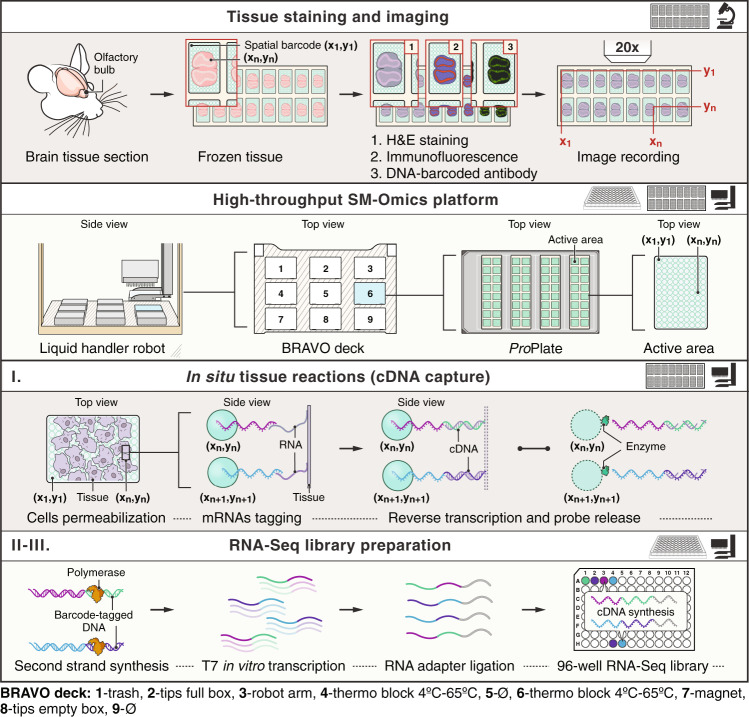
SM-Omics. Overview of approach. SM-Omics approach combines automated imaging of H&E, IF stained or tissue sections stained with DNA-barcoded antibodies with high-throughput liquid handling to create spatially resolved RNA-Seq and/or antibody-seq libraries. The RNA-Seq protocol consists of three main steps. (I) in situ reactions on a ST slide that include tissue permeabilization, capture of mRNAs on the spatial array followed by a reverse transcription reaction in solution. The transcribed material is then collected and a two-step library preparation protocol (II–III) is run in standard 96-well plates.

We developed the SM-Omics platform for either automated Spatial Transcriptomics alone, or, in combination with fluorescently or DNA-barcoded antibodies to simultaneously measure spatial profiles of RNAs and proteins. Briefly, in SM-Omics, after tissue staining for traditional hematoxylin and eosin histology (H&E), IF or using DNA-barcoded antibodies, glass slides are loaded into the SM-Omics platform, where, using a liquid handler robot, cells are permeabilized, mRNAs and/or antibody barcodes are spatially tagged and converted into a sequencing-ready library (Fig. 1). The automated process consists of three main parts with designed stopping points to either store the processed material or load required reagents for the upcoming reactions. The first step consists of all in situ enzymatic reactions on the SM-Omics slide, including tissue permeabilization after staining and reverse transcription with simultaneous release of spatial capture probes (Fig. 1I). Each such in situ run holds up to 4 slides with tissues, with the number of active areas with spatial probes per slide ranging from one to 16 per slide. The second and third steps consist of RNA-Seq library preparation in standard 96 well plates, where the user can choose to run between 1 and 96 libraries in parallel in 8-step increments with adjusted library consumable usage to alleviate costs. The input to these is in situ spatial tissue cDNA or DNA-barcoded antibody tags captured from glass slides in the first step, which are then processed to amplify cDNA using a T7 in vitro transcription approach (for cDNA) or standard PCR amplification (for DNA-barcoded antibody tags), followed by a final conversion of the amplified RNAs into sequencing-ready libraries (Fig. 1II, III).

SM-Omics introduces four key enhancements compared to ST: (1) automation, requiring minimal user intervention; (2) throughput, allowing processing of 96 libraries in a 2-day cycle; (3) enhanced quality, reflected by higher complexity RNA-Seq libraries and (4) combining RNA-Seq measurements with multiplex protein measurements including IF staining and antibody-barcoding strategies. To process the generated data efficiently, we also developed SpoTteR, a fast and fully automated end-to-end image registration method (Methods). We first describe the core approach in the context of spatial RNA measurements (Fig. 1, II, III), and then its extension to include spatial antibody-based protein measurements.

### In situ SM-Omics RNA-seq

To test the performance of SM-Omics for spatial transcriptomics, we assessed the feasibility, reproducibility and efficiency of RNA data in two key steps, testing on the mouse main olfactory bulb (MOB) and mouse cortex: (1) in situ tissue reactions (cDNA capture) and (2) cDNA library preparation for RNA-Seq.

SM-Omics had enhanced performance in terms of in situ reactions compared to standard ST, with minimal lateral diffusion and comparable and reproducible cDNA signal intensity. Specifically, we first ran in situ reactions on the glass surface in optimization mode, where cDNA molecules are in situ fluorescently labeled to create a spatial cDNA footprint^36^ (Supplementary Fig. 1a). We compared the localized cDNA footprint to the histological H&E pattern and measured the lateral tissue permeabilization effects. This provides an optimal set of parameters needed to successfully run tissue-specific reactions and to ensure minimal lateral cross-talk between adjacent spatial measurements. Testing on the adult mouse cortex (Supplementary Fig. 1b–e') showed that SM-Omics resulted in no mixing of material between spatial measurements with no lateral diffusion (mean −0.06 µm ± 0.51 µm sd), which is 4x weaker lateral diffusion signal than in ST performed on adjacent tissue sections (two-sided Wilcoxon’s rank-sum test, *p*-value ≤ 0.05, Supplementary Fig. 1f, g), and 30x weaker diffusion signal compared to previous reports^24,36,47^. Moreover, the signal intensity of the fluorescent cDNA footprint was highly reproducible within and between SM-Omics runs: there were no significant differences (two-sided Wilcoxon’s rank-sum test, *p*-value > 0.05) between the cDNA signal intensities from adjacent adult mouse MOB tissue replicates on a single glass slide (*n* = 3), single run (*n* = 3) or separate runs (*n* = 3) (Supplementary Fig. 2).

To process the generated data efficiently, we also developed SpoTteR, a fast and fully automated end-to-end image registration method. SpoTteR automatically downscales images and reconstructs barcode spots positions using iterative spot detection and grid fitting (Methods), accounting for common imaging artifacts, such as uneven tissue coloration or pipetting bubbles. SpoTteR then registers tissue coordinates through a masking process to produce a gene-by-barcode matrix overlaid on top of morphological features (Supplementary Fig. 3). Compared to manual and semi-automated image registration approaches^48^, SpoTter is up to 14X faster with low false discovery rates (FP 3.54% and FN 1.18%, *vs*. >15% of grid spots as FNs in other approaches^48^), when applied to images of human lung cancer, human arthritis and mouse colon data (Supplementary Fig. 3b, Supplementary Fig. 4).

**Fig. 2:**
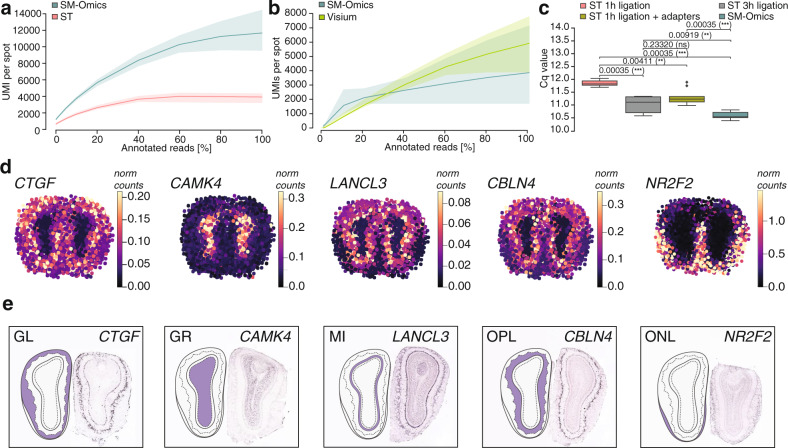
SM-Omics performance. **a**, **b** Sensitivity of spatial gene expression measurements. Mean number of unique molecules detected (*y* axis) at different proportions of annotated reads (*x* axis) in **a** SM-Omics (blue, *n* = 3) and ST (red, *n* = 3) and **b** SM-Omics (blue, *n* = 3) and Visium (green, *n* = 3). Shaded areas: 95% confidence intervals. Colored line: mean of summarized library values (*n* = 3) per condition. **c** Performance of automated spatial library preparation reactions. Impact of ligation reaction times and adapter concentrations on quantitative concentrations (Cq) values for automated prepared libraries (*n* = 9). Cq values were measured at Fluorescent unit 10,000. Statistical significance (two-sided Wilcoxon’s rank-sum test) markings are displayed: 0.05 < *p* ≤ 1 (ns), 0.001 < *p* ≤ 0.01 (**), 0.0001 < *p* ≤ 0.001 (***). Center black line, median; color-coded box, interquartile range; error bars, 1.5x interquartile range; black dots; outliers. Individual reaction conditions are detailed in Methods. **d**, **e** Spatial gene expression. **d** Examples of SM-Omics spatial gene expression patterns (normalized expression shown in color scale) detected in each of the major histological regions in the MOB of an adult mouse brain and **e** corresponding in situ hybridization images from ABA (Image credit: Allen Institute for Brain Science, Methods) for the same genes as in **d** with illustrated and highlighted region annotation patterns. Annotated region abbreviations: GL (glomerular layer), GR (granular cell layer), MI (mitral layer), OPL (outer plexiform layer) and ONL (olfactory nerve layer).

Using the SM-Omics end-to-end toolbox (Fig. 1) we prepared and sequenced SM-Omics (*n* = 3) high quality RNA-Seq libraries (Supplementary Data 1) from the MOB of the adult mouse brain, and compared them to standard ST (*n* = 3) libraries at the same sequencing depth (by down-sampling). SM-Omics RNA-Seq libraries were more sensitive than ST, with a 3.2-fold higher number of unique protein-coding genes and a 3.6-fold higher number of unique transcripts (UMIs) present in the data (Wald’s test, *p*-value ≤ 0.05, Methods, Supplementary Fig. 5a). Per spatial measurement, SM-Omics detected 2.5-fold more unique genes (3748 ± 562) and 3.5-fold more unique transcripts (11,261 ± 2273 UMIs) than ST (1485 ± 185 genes; 3188 ± 513 UMIs) (Wald’s test, *p* ≤ 0.05, Methods, Fig. 2a, Supplementary Fig. 5b). SM-Omics exhibited an increase on average (*n* = 3) in the number of transcripts captured in most of the annotated morphological regions compared to ST (Wald’s test, *p*-value ≤ 0.05, Methods, Supplementary Fig. 5c, Supplementary Data 2) and performed comparably to newer array designs (*n* = 3) (Visium, 10x Genomics, effect size = 2.11 and 1.38, for genes and UMIs respectively, *p*-value > 0.05, Wald’s test) (Fig. 2b, Supplementary Fig. 5d, e). This increased efficiency in SM-Omics, as reflected in the number of genes and UMIs detected per (x, y) coordinate, was due to several optimizations in library preparations. First, we introduced simultaneous release of barcoded primers and captured mRNA molecules (Methods) from the glass surface which also decreased total in situ processing time from ~1.5 days to ~6 h. Second, we improved the efficiency of library preparation reactions, by increasing the amount of sequencing adapters and reaction time for adapter ligation to the template (Methods, two-sided Wilcoxon’s rank-sum test, *p* ≤ 0.05) (Fig. 2c).

To further test SM-Omics RNA-Seq on challenging tissues, we optimized in situ reaction conditions for mouse colon and colorectal cancer models, and found strong spatial fluorescent patterns in these tissues (Supplementary Fig. 6a, b). Moreover, SM-Omics (*n* = 3) outperformed ST (*n* = 3) in library metrics per spatial measurement in the cancer model. SM-Omics detected significantly higher (Wald’s test, *p*-value ≤ 0.05, Supplementary Fig. 6c) numbers of genes and UMIs (5086 ± 121 genes and 16,250 ± 922 UMIs) compared to ST (2733 ± 492 genes and 5128 ± 1304 UMIs).

We also compared SM-Omics and ST in terms of detecting spatial expression patterns. We used Splotch^32,49^ to align an expanded dataset of 18 replicate MOB tissue sections and generate posterior spatial gene expression estimates. We confirmed that region-enriched and upregulated genes were present in the major spatial layers (Methods) of the MOB compared to the reference Allen Brain Atlas (ABA) data^50^ (Supplementary Fig. 7a, b). While known gene patterns detected as layer-enriched agreed between SM-Omics and ST (Supplementary Fig. 7c–f), SM-Omics’ overall specificity was higher (Supplementary Fig. 7a) and gene expression values per region were more highly correlated between SM-Omics and ABA (Spearman’s ρ = 0.90, *p*-value ≤ 0.0001, Supplementary Fig. 7g) than between ST and ABA (Spearman’s ρ = 0.71, *p*-value ≤ 0.005, Supplementary Fig. 7g). Compared to Visium, SM-Omics exhibited comparable regional metrics, with both methods showing enrichment of regionally expressed genes in the appropriate spatial layers of the mouse brain cortex, and high correlation to expression levels in the ABA (Supplementary Fig. 8a, b), with similar regional sensitivity for both SM-Omics and Visium (Supplementary Fig. 8c). This increased sensitivity (*vs*. ST) at the same sequencing depth (by down-sampling, Methods), allowed us to reproducibly measure the spatial gene expression of newly detected targets, otherwise not detected by standard ST, such as *CTGF* in the glomerular layer (GL) and *CAMK4* in the granular cell layer (GR), both implicated in impairments in retention of long-term memory^51^ and acting as targets of protein aggregation in models of Alzheimer’s disease^52^, as well as *LANCL3* in the mitral layer (MI), *NR2F2* in the olfactory nerve layer (ONL) and *CBLN4* in the outer plexiform layer (OPL) (Fig. 2d, e). Identifying and quantifying these additional genes using SM-Omics’ increased sensitivity should help discover novel biological targets as well as pursue hypothesis-driven research.

### Spatial transcriptomics with antibody-based immunofluorescence

**Fig. 3:**
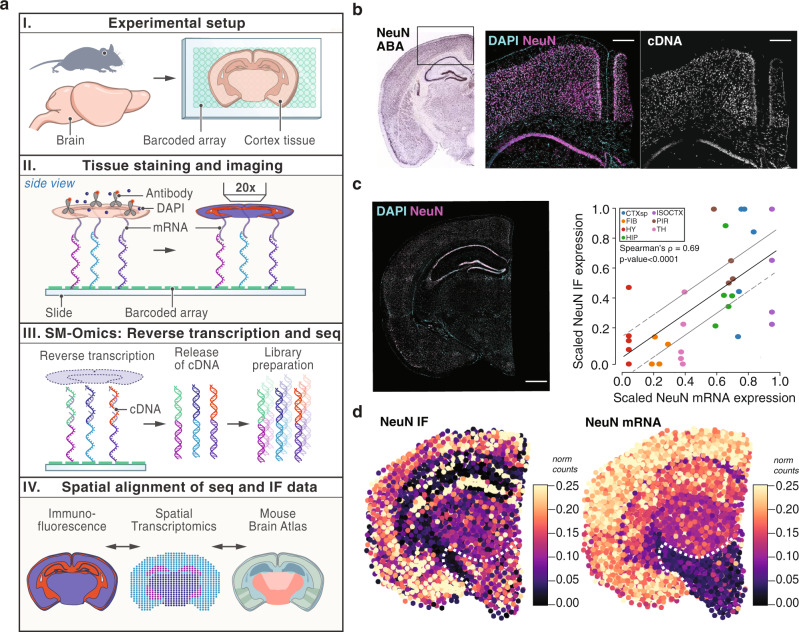
Spatial RNA-Seq and immunofluorescence highlights tissue specific expression patterns in the mouse brain cortex. **a** Experimental setup. Tissue sections are placed on the spatial array (I), stained for nuclear and corresponding antigen targets, imaged for IF signals (II) and SM-Omics libraries created (III). Spatial gene and antibody expression data are processed and compared to the reference ABA atlas (IV). **b** Combined antibody IF and spatial transcriptomics in situ measurements. ABA in situ hybridization reference image (left) with NeuN staining neuronal nuclei with marked isocortex area (rectangle). Mouse brain isocortex tissue (*n* = 3) stained for DAPI (middle; cyan) and NeuN IF (middle; purple) and corresponding fluorescent gene activity cDNA footprint (right; white). Scale bar; 200 µm. **c**, **d** Performance of combined antibody IF and spatial transcriptomics measurements. **c** NeuN immunofluorescence (stained tissue section, left; scale bar 800 µm; and y axis; mean scaled signal, right) and mRNA in situ measurements (*x* axis, scaled normalized expression, right) per tissue section (*n* = 5, Methods) in each of seven regions (color code) in SM-Omics. Black line: linear regression with respective standard deviations (gray lines). **d** Antibody IF signals (normalized and scaled expression shown in color scale, NeuN IF; left) and mRNA expression (normalized and scaled expression shown in color scale, NeuN mRNA; right) aggregated in SM-Omics-like spots. White dashed lines: hypothalamus region. Annotated region abbreviations: CTXsp (cortical subplate), FIB (fiber tracts), HY (hypothalamus), HIP (hippocampal formation), ISOCTX (isocortex), PIR (piriform areas) and TH (thalamus).

We next developed a protocol that combined antibody-based IF with spatial transcriptomics (Fig. 3a, Methods). Localized cDNA footprints after nuclear (DAPI) and IF staining of the tissue (Fig. 3b, Supplementary Fig. 9a) showed that mRNAs were laterally diffusing only 0.16 ± 1.21 μm outside of the nucleus, again indicating minimal lateral cross-talk between adjacent spatial measurements. We next created SM-Omics mouse brain cortex libraries following immunostaining with an antibody against the brain protein NeuN, which is highly expressed in most neuron nuclei (Fig. 3c). Library complexities, signal specificity and RNA expression patterns were similar to those in standard (H&E stained) SM-Omics RNA-Seq measurements and in ABA^50^ (Supplementary Fig. 9b–d), confirming that our protocol for simultaneous IF and transcriptome measurements provided high-quality mRNA data. Next, comparing the antibody IF signals and corresponding RNA expression (Fig. 3c), there was significant correlation between NeuN mRNA and aggregated protein expression (Spearman’s ρ = 0.69, *n* = 5, *p*-value ≤ 0.0001, Fig. 3c) across all major regions in the mouse brain cortex. Notably, in some regions (e.g., hypothalamus) RNA expression was low but protein expression was substantial (Fig. 3d). This may be due to either a biological difference, or to the differences in sensitivity and saturation of RNA-Seq *vs*. IF. Furthermore, while throughput in antibody-based IF is limited and imaging data and mRNA data have different noise characteristics (Supplementary Data 2), it provides a fast alternative to traditional H&E staining as well as adds quantitative protein information at single-cell resolution to any spatial array design.

### An all-sequencing-based approach for spatial multi-omics

**Fig. 4:**
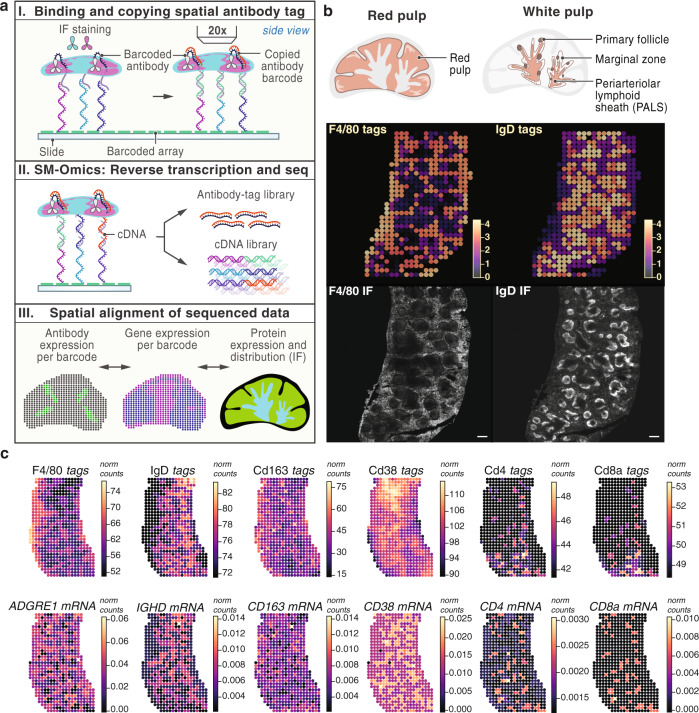
Spatial RNA-Seq and protein profiling with DNA-barcoded antibodies in mouse splenic tissue. **a** Experimental setup. SM-Omics approach combines automated imaging of IF antibody stained tissue sections, tagging antigens spatially in situ using DNA-barcoded antibodies and capturing mRNA on a spatially barcoded poly(d)T array. Frozen tissue sections are placed on a SM-Omics array, tissues stained with both IF and DNA-barcoded antibodies, imaged and in situ copying reactions performed and at the same time as cDNA is made (I). Then, both the antibody tags and cDNAs are used in the library preparation reactions and sequenced (II). Finally, spatial IF, antibody tag and gene expression patterns can be evaluated (III). **b** Performance of combined antibody IF and DNA-barcoded antibody signal measurements. Splenic tissue illustration of red and white pulp structures (top) followed by spatial expression profiles of sequenced antibody tags (middle; normalized expression shown in color scale) as well as IF images (bottom) in splenic tissue for F4/80 staining red pulp macrophages and IgD staining marginal zone B cells in the white pulp (*n* = 7). Scale bar (bottom) denotes 300 µm. **c** Performance of combined DNA-barcoded antibody signal and spatial transcriptomics measurements. Spatial expression profiles (normalized expression shown in color scale) for a 6-plex SM-Omics reaction with F4/80, IgD, Cd163, Cd38, Cd4 and Cd8a DNA-barcoded antibody-based expression in the top panel (tags) and respective gene expression shown in the bottom panel (mRNA).

Finally, we introduced an antibody DNA-barcoding system^5^ compatible with spatial transcriptomics to increase multiplexing capacities otherwise limited with spectral overlap in imaging approaches (Fig. 4a). We tagged each of 6 antibodies^5^ with an amplification primer and an individual barcode tag followed by a poly(d)A sequence for capture on a poly(d)T spatially barcoded array (Methods). We used a similar tissue staining protocol as that for IF, where the tissue was first in situ fixed with paraformaldehyde to ensure specific antigen coupling, followed by antibody staining, tissue permeabilization and SM-Omics library preparations (Fig. 4a). To benchmark our approach, we incubated adult mouse spleen tissue sections with both a fluorescently labeled antibody and a DNA-barcoded antibody (i.e. antibody tag), allowing us to simultaneously validate and directly compare both detection methods. We imaged the fluorescently labeled epitopes prior to any in situ enzymatic reactions on the array surface, coupled the antibody tags to the spatial array, such that they were copied into a stable covalent complex, while mRNA was spatially captured and transcribed on the array (Fig. 4a).

We first tested a two-antibody cocktail targeting F4/80 and IgD (Fig. 4b), aimed to stain distinct spatial niches in the mouse spleen: splenic red pulp macrophages and marginal zone B cells in the white pulp, as previously described^19^. We obtained high quality antibody tag (mean ± sd 142 ± 15 UMIs per SM-Omics measurement; *n* = 7) and cDNA libraries (1375 ± 181 UMIs per SM-Omics measurement, *n* = 3), with highly specific antibody tag patterns (Fig. 4b) that were well-correlated to the corresponding IF intensities across all major splenic regions (Supplementary Fig. 10a, on average 76%, *p* ≤ 0.0001). RNA and antibody tag levels were in agreement for IgD (Spearman’s ρ = 0.73, *n* = 3, *p*-value ≤ 0.05 across all spatial measurements), and less so for F4/80 (Spearman’s ρ = 0.65, *n* = 3, *p*-value ≤ 0.05 across all spatial measurements) (Supplementary Fig. 10b).

Finally, an SM-Omics experiment with six validated^53^ DNA-barcoded antibodies targeting F4/80, IgD, Cd163, Cd38, Cd4, and Cd8a (Supplementary Fig. 10c), spanning different levels of expression and spatial patterns, successfully combined spatial transcriptomics and protein estimates in a highly multiplexed manner (Fig. 4c, Supplementary Fig. 10d). Cd4 and Cd8 proteins (by antibody signal) and their corresponding mRNAs were spatially localized in the PALS zone (Spearman’s ρ = 0.59, *n* = 3, *p*-value ≤ 0.05), whereas IgD and Cd38 protein and mRNA were enriched in the B follicles (Spearman’s ρ = 0.66, *n* = 3, *p*-value ≤ 0.05), with protein expression high in all white pulp areas (Supplementary Fig. 10d). F4/80 protein and mRNA were localized to the red pulp, but the corresponding mRNA (*ADGRE1*) was also enriched in the marginal zone (Supplementary Fig. 10d). Finally, Cd163 was differentially expressed, as expected, in the red pulp, however, *CD163* mRNA was high, apart from the red pulp zonations in PALS as well, while protein levels were not detected at significant levels in that same tissue area (*p*-value > 0.05, Supplementary Fig. 10d).

## Discussion

SM-Omics is an efficient and automated workflow for combined and spatially resolved transcriptomics and antibody-based protein measurements, adaptable to new array versions and designs. SM-Omics provides a more detailed molecular high-plex multi-omics characterization of tissues in situ and is a high-throughput automated system for quantifying the spatial transcriptome and antibody-based protein detection, by either IF or using DNA-barcoded antibodies. Compared to approaches with similar array design versions (Visium, 10X Genomics), SM-Omics provides an automated workflow that is not limited to performing a small number of high-resolution spatial IF measurements but further extends the combined spatial transcriptomics and spatial antibody-based protein measurements into a scalable all-sequencing based technology. Using a 6-plex proof-of-concept antibody SM-Omics reaction, we confirmed that SM-Omics is a robust system that can reconstruct specific cell associations across morphological layers^54,55^, and characterize tissue niches in combination with antibody staining, which provide higher resolution views independently of or in combination with spatial transcriptomics patterns.

SM-Omics can be enhanced in the future in several ways, including demonstrating higher multiplex for protein detection (similarly to CITE-seq^5^), automating tissue sectioning workflows, increasing the resolution of spatial measurements (to achieve that of recent spatial RNA-seq approaches^26,56–58^) and furthering work on integrating robust image registration and IF pipelines to aid in interpreting combined signals from different modalities created with SM-Omics. Moreover, SM-Omics is currently limited to frozen tissues (whereas many clinical samples are FFPE) and to lower resolution arrays, and future studies can tackle those to extend its applicability and enhance its resolution, respectively. Finally, while current costs of commercial spatial arrays might be limiting (Supplementary Data 1), high throughput processing should motivate economies of scale.

SM-Omics automation on a widely-used platform enables use of appropriate study design while minimizing technical variation, and allowing broad adoption. Additionally, even if only used as a spatial transcriptomics library preparation system, its 96-plex throughput outperforms previous automated protocol designs by 6–8 fold^34,35^. SM-Omics does not rely on any customized liquid handling microfabrication, uses commercially, widely-available liquid handlers and reagents with minimum preparation time per run (~30 min), has an end-to-end image-integrated data registration pipeline and is readily deployable to the wide scientific community.

## Methods

### Ethical statement

All work involving C57BL/6 J mice was performed under specific-pathogen-free conditions and the guidelines of the Division of Comparative Medicine, in accordance with the Institutional Animal Care and Use Committees (IACUC) relevant guidelines at the Broad Institute of Harvard and MIT, and consistent with the Guide for Care and Use of Laboratory Animals, National Research Council, 1996 (institutional animal welfare assurance no. A4711-01), with protocol 0122-10-16.

### Bravo system requirements

Bravo Automated Liquid Handling Platform (Agilent Technologies, USA) was equipped with a 96LT pipetting head (G5498B#042, Agilent Technologies, USA) and two Peltier thermal stations (CPAC Ultraflat HT 2-TEC, #7000166 A, Agilent Technologies, USA) with PCR adapter having a mounting frame at positions 4 and 6 on the Bravo Deck and connected to an Inheco MTC Controller. On position 7, we recommend the MAGNUM FLX™ Enhanced Universal Magnet Plate (#A000400, Alpaqua, USA) to serve for magnetic bead-based clean ups. In addition, a BenchCel NGS Workstation (Front-load rack at 660 mm height) and BenchCel Configuration Labware MiniHub (option #010, Agilent Technologies, USA) were included in the automation platform setup. In case in situ reactions were performed, the PCR adapter was removed from position 6 to be replaced with Aluminum Heat Transfer Plate (#741I6-GS-4, V&P Scientific, Inc, USA). This liquid handling setup enables running in situ reactions using the ProPlate Multi-Array slide system (GraceBioLabs, USA), where 64 reactions can be run in parallel using the standard 96LT pipetting head. Note that every third column in the 96-tip pipette box needs to be removed when using the ProPlate Multi-Array system with standard Agilent Bravo pipetting instrumentation. All library preparation reactions are run in a maximum 96-well mode, however lower throughput adjustments are predefined as 8-sample increments and easily loaded in our automated SM-Omics settings. Further details in the SM-Omics protocol sections below, and at: https://github.com/klarman-cell-observatory/sm-omics/tree/master/SM_Omics_v.B1.0.2.

### Sample collection and cryosectioning

All work involving C57BL/6 J mice was performed under specific-pathogen-free conditions and the guidelines of the Division of Comparative Medicine, in accordance with the Institutional Animal Care and Use Committees (IACUC) relevant guidelines at the Broad Institute of Harvard and MIT, and consistent with the Guide for Care and Use of Laboratory Animals, National Research Council, 1996 (institutional animal welfare assurance no. A4711-01), with protocol 0122-10-16. A small piece of freshly collected tissue (~25–50 mg, about 5 × 5 mm) was placed on a dry and sterile Petri dish, which was placed on top of wet ice. The tissue was then very gently moved using forceps and placed on another dry part of the Petri dish to ensure little liquid was present around the tissue. The bottom of a cryomold (5 × 5 mm, 10 × 10 mm or 25 × 20 mm) was filled with pre-chilled (4 °C) OCT (Tissue-Tek; Sakura Finetek, USA) and the tissue transferred with forceps into the OCT-prefilled mold. The entire tissue surface was covered with pre-chilled OCT. The mold was then placed on top of dry ice and allowed the tissue to freeze for up to 5 min until OCT has turned completely white and hard. The tissue cryomolds were stored at −80 °C until use. For cryosectioning, the ST slide and the tissue molds first reached the temperature of the cryo chamber. The OCT-embedded tissue block was attached onto a chuck with pre-chilled OCT and allowed to freeze ~5–10 min. The chuck was placed in the specimen holder and adjusted the position to enable perpendicular sectioning at 10 µm thickness. Sections were gently transferred to a ST array^24^ and then the back side of the slide was warmed ~10–15 s with a finger. ST slides with tissue sections on top could be stored at −80 °C for up to 6 days.

### Tissue fixation and H&E staining

The ST slide with the tissue section was warmed to 37 °C for 1 min on a thermal incubator (Eppendorf Thermomixer Option C, Germany). The tissue was then covered with 4% formaldehyde (Sigma-Aldrich, USA) in 1X PBS (Thermo Fisher Scientific, USA) for 10 min at room temperature (RT). The whole slide was then washed in 1X PBS in a vertical orientation to be placed back on a horizontal place for drying. 500 µl isopropanol covered the tissue and ensured drying. The slide was put into an EasyDip Slide Jar Staining System (Weber Scientific) holder and the same system used for H&E staining. Five ~80 ml containers were prepared with Dako Mayers hematoxylin (Agilent, USA), Dako Bluing buffer (Agilent, USA), 5% Eosin Y (Sigma–Aldrich, USA) in 0.45 M Tris acetate (Sigma–Aldrich, USA) buffer at pH 6 and two jars with nuclease-free water (ThermoFisher Scientific, USA). The slide rack was fully immersed in hematoxylin for 6 min and then washed by dipping the slide rack in a nuclease-free water jar 5 times following another destaining wash by dipping the slide rack in 800 mL nuclease-free water for 30 times. The slide rack was put into the Dako bluing buffer and incubated for 1 min. The slide was again washed by dipping the rack 5 times in the second nuclease-free water jar. The slide rack was finally put into the eosin and incubated for 1 min to be washed by dipping the rack 7 times in the second water jar. The slide was removed from the rack to allow it to dry.

### Tissue fixation and IF staining

The ST slide with the tissue section was warmed to 37 °C for 4 min on a thermal incubator (Eppendorf Thermomixer Option C, Germany) and in situ fixed and washed as described above. The slide was then mounted in the plastic slide holder (ProPlate Multi-Array slide system; GraceBioLabs, USA) compatible with the Aluminum Heat Transfer Plate (#741I6-GS-4, V&P Scientific, Inc, USA) on position 6 on the Bravo deck. All following antibody incubations were performed at 4 °C. First, the tissues were blocked with the TruStain FcX™ PLUS (anti-mouse CD16/32, Biolegend, USA) antibody (1:100 dilution) in 0.5% Triton X-100 (Sigma-Aldrich, USA) for mouse brain tissues and 1× perm/wash buffer (ThermoFisher Scientific, USA) for splenic tissues. This simultaneous blocking and permeabilization step lasted for 30 min. Next, the slide was washed 3× with 1× PBS (ThermoFisher Scientific, USA). After discarding the last wash, the slides were incubated with 1× PBS for 2 min. Then, antibodies were added at 1:100 dilution for 90 min. The complete list of antibody clones and suppliers is available in Supplementary Data 3. The slide was again washed in the same fashion and counterstained with DAPI (Sigma–Aldrich, USA) diluted 1:1000 in 0.5% Triton X-100 (Sigma–Aldrich, USA) for 5 min. In case the reactions were performed on a SM-Omics array and not a mock polyd(T) array, the DAPI reaction was also supplemented with a Cy3 labeled anti-frame DNA probe (5′-Cy3-GGTACAGAAGCGCGATAGCAG-3′, IDT, USA) at 10 nM concentration. In case DAPI counterstaining was not used, the step was skipped. This was followed by another wash cycle. The slides were then air dried and mounted with 85% glycerol prior to imaging.

### Tissue fixation and DAPI-only staining

Similarly to performing *Tissue fixation and IF staining*, tissue sections were attached to slides and in situ fixed. The slide was then mounted in the plastic slide holder (ProPlate Multi-Array slide system; GraceBioLabs, USA) and all reactions performed at 4 °C. Tissues were first incubated with 0.5% Triton X-100 (Sigma–Aldrich, USA) for 25 min. Next, the slide was washed 1x PBS (ThermoFisher Scientific, USA) and the tissue stained with DAPI (Sigma–Aldrich, USA) diluted 1:1000 in 0.5% Triton X-100 (Sigma–Aldrich, USA) for 15 min. If the reactions were performed on a SM-Omics array and not a mock polyd(T) array, the DAPI reaction was also supplemented with a Cy3 labeled anti-frame DNA probe (5′-Cy3-GGTACAGAAGCGCGATAGCAG-3′, IDT, USA) at 10 nM concentration in order to facilitate image registration to the SM-Omics array coordinates. This was followed by another wash cycle. The slides were then air dried and mounted with 85% glycerol prior to imaging.

### Automated imaging

Images of stained H&E tissue sections on the ST slides were taken using a Metafer Vslide scanning system (MetaSystems, Germany) installed on an Axio Imager Z2 microscope (Carl Zeiss, Germany) using an LED transmitted light source and a CCD camera (BF scanning). All images were taken with the A-P 10x/0.25 Ph1 objective lens (Carl Zeiss, Germany). For fluorescent scanning, a PhotoFLuor LM-75 lightsource (89North, USA) was used in combination with a Plan-APOCHROMAT 20x/0.8 objective (Carl Zeiss, Germany). A configuration program was made to enable automatic tissue detection, focusing and scanning on all ST arrays present on a glass slide. In short, tissue detection was based on contrast as compared to normalized background in all channels. Upon finding maximum contrast in a 12-step spiral-like search window field of view (FOV) pattern, the automated focal alignment in every second of each FOV (4096 × 3000 px) was initiated. The alignment search considered the maximum contrast z-position as in-focus using 5 µm stage intervals (*n* = 19 focal planes). The BF scanning of the predefined ST array areas was done in a total of 48 FOVs and ~30 s in 3 channels (RGB); or epifluorescent scanning of 228 FOVs and ~6 min for 3 fluorescent channels. Images were stitched using 60 µm overlap and linear blending between FOVs with the VSlide software (v1.0.0) and then extracted using jpg compression. Multiple ST slides can be processed in the same manner without any user input for a total of 6 min processing time per H&E stained slide (3 channels) or 45 min for fluorescently stained slide (3 channels), including image stitching.

### Microarray design and production

Both for quality control experiments and library preparation, the Codelink amine activated slides (#DN01-0025, Surmodics, USA) were exposed with polyadenylated oligonucleotides (IDT, USA) and microarray production proceeded as according to manufacturer’s instructions (Surmodics, USA). The surface oligonucleotides are presented here for clarity:

([AmC6]UUUUUGACTCGTAATACGACTCACTATAGGGACACGACGCTCTTCCGATCT[18nt]NNNNNNN[20 T]VN).

This chemistry design enabled covalent linking upon binding to the Codelink slide surface. For library preparation slide production, 33 μM spatially barcoded oligonucleotides (IDT, USA) were deposited as 100pL droplets onto Codelink slides as suggested by the manufacturer (Surmodics, USA). This resulted in about ~200 million copies of the oligonucleotide per spatial spot. Array printing was performed by ArrayJet LTD (Scotland, UK) according to the ArrayJet Spider system requirements. Each library preparation slide active area had a total of 1,007 spatially barcoded positions distributed over a ~42 mm^2^ area. Each spatially barcoded ST spot had a diameter of 100 μm, with a center-to-center distance of 200 μm between the spots.

### SM-Omics automation

The SM-Omics protocol is divided into three main parts. The first part (1) processes all in situ reactions on a ST slide: tissue pre-permeabilization, permeabilization, reverse transcription with or without the release of the spatial capture probes and tissue removal. This material is collected to a standard 96-well PCR microplate (Eppendorf, Germany) and all of the following reactions (protocols 2 and 3) are run in 96-well plates. The second protocol (2) contains second strand synthesis reaction, cDNA bead purifications and T7 in vitro transcription. The third protocol (3) includes aRNA adapter ligation, bead purifications and second cDNA synthesis. The material is then quantified using a standard qPCR protocol and the libraries accordingly indexed for Illumina sequencing.

### Reference material preparation

In order to test reproducibility of library preparation reactions, we prepared reference material as input. 7.5 µg of universal mouse reference RNA (#740100, Agilent Technologies, USA) was fragmented using NEBNext Magnesium RNA fragmentation module (NEB, USA) for 1 min at 94 °C. The sample was purified with a MinElute Cleanup kit (Qiagen, Germany) according to manufacturer’s instructions and RNA concentration and size were assessed using a Qubit RNA HS kit (ThermoFisher Scientific, USA) and Bioanalyzer Pico 6000 kit (Agilent Technologies, USA), respectively. ~2 µg of fragmented RNA was incubated with either 3.3 µM custom hexamer primer (GACTCGTAATACGACTCACTATAGGGACACGACGCTCTTCCGATCTNNNNNNNN, T7handle_IlluminaAhandle_hexamer) or poly(d)T primer (T7handle_IlluminaAhandle_hexamer_20TVN) in the presence of 0.8 mM dNTP (ThermoFisher Scientific, USA) at 65 °C for 5 min. First strand reverse transcription was performed with a final concentration of 1X First Strand Buffer, 5 mM DTT, 2U/µl RNaseOUT and 20U/µl of Superscript III (all from Thermo Fisher Scientific, USA). The reaction was incubated at 25 °C for 10 min (when using hexamer priming), followed by 50 °C for 1 h and 70 °C for 15 min or 50 °C for 1 h and 70 °C for 15 min for poly(d)T priming. The reaction was purified with AMPure XP beads (Beckman Coulter, USA) at a beads/DNA ratio of 0.8:1. The concentration of the material was measured on a Qubit RNA HS kit (ThermoFisher Scientific, USA) and diluted in EB (Qiagen, Germany). A release mixture of ~100 ng (hexamer priming) or ~200 ng (poly(d)T priming) first strand cDNA, 1X Second strand buffer (ThermoFisher Scientific, USA), 0.2 µg/µl BSA and 0.5 mM dNTP (ThermoFisher Scientific, USA) was used to test all library preparation reactions. Hexamer primed cDNA was used to test the reproducibility and poly(d)T primed cDNA was used to test adapter concentrations and ligation time.

### In situ SM-Omics protocol (1)

Tissue-stained ST slides we provided as input. The ST slide was attached into the ProPlate Multi-Array slide system (GraceBioLabs, USA), with up to four ST slides fitted. The ProPlate Multi-Array system was then fixed in position by Aluminum Heat Transfer Plate (VP 741I6-GS-4, V&P Scientific, Inc, USA) on the Agilent Bravo deck. The protocol started with tissue pre-permeabilization (30 min at 33 °C) with addition of 120 µl reagent per well of exonuclease I buffer for brain samples (NEB, USA) or 120 µl reagent per well of collagenase I (200U) in 1x HBSS (both from Thermo Fisher Scientific, USA) for colorectal samples. For spleen sections, the pre-permeabilization step was skipped. For complete removal of the reagents and wash solutions from the subarrays all of the robotic dispensing and aspiration steps took place in all four corners of the square wells. Pre-permeabilization reagent removal was followed by a 180 µl wash in 0.1X Saline Sodium Citrate (SSC, Sigma–Aldrich, USA) at 33 °C. Next, tissue permeabilization was done using 75 µl 0.1% pepsin (pH 1, Sigma–Aldrich, USA) at 33°C for 10 min (mouse brain) and 15 min (colorectal cancer) and for 60 min (spleen) 75 µl 0.1% pepsin prepared at pH 2.5 in Tris-HCl (Sigma-Aldrich, USA). After a 180 µl 0.1X SSC wash at 33 °C, in situ cDNA synthesis reaction was performed by the addition of 75 µl RT reagents: 50 ng/µl actinomycin D (Sigma–Aldrich, USA), 0.5 mM dNTPs (Thermo Fisher Scientific, USA), 0.20 µg/µl BSA, 1 U/µl USER enzyme (both from NEB, USA), 6% v/v lymphoprep (STEMCELL Technologies, Canada), 1 M betaine (#B0300-1VL, Sigma-Aldrich, USA), 1X First strand buffer, 5 mM DTT, 2 U/µl RNaseOUT, 20 U/µl Superscript III (all from Thermo Fisher Scientific, USA). The reactions were sealed with 70 µl of white mineral oil Drakerol#7 (Penreco, USA). Incubation at 30 °C was performed for a minimum of 6 h, after which 70 µl of the released material was collected in a new 96-well PCR plate (Eppendorf, Germany). When a Cy3 fluorescent cDNA activity print was needed for tissue optimization, the 75 µl in situ cDNA reaction mix was as follows: 50 ng/µl actinomycin D (Sigma-Aldrich, USA), 0.20 µg/µl BSA (NEB, USA), 1X M-MuLV buffer, 5 mM DTT, 2U/µl RNaseOUT, 20U/µl M-MuLV (all from Thermo Fisher Scientific, USA), 4 µl dNTP mix (dATP; dGTP and dTTP at 10 mM and dCTP at 2.5 mM) and 2.2 µl Cy3-dCTPs (0.2 mM, Perkin Elmer, USA).

### In situ manual ST protocol

The manual ST in situ protocol was performed as described in Salmén et al.^47^. The protocol is, if not mentioned below, identical to the robotic protocol except as further described. Tissue-stained ST slide was attached in an ArrayIT hybridization chamber (ArrayIT, CA). All incubations took place on an Eppendorf Thermocycler R (Eppendorf, Germany), and reactions were covered with Microseal ‘B’ PCR Plate Seals (Biorad, CA) to avoid evaporation. Pre-permeabilization and washes were performed with 100 µl reagent at 37 °C and the in situ cDNA synthesis reaction was run without the USER enzyme, lymphoprep and betaine, at 42 °C. The manual protocol then encompassed tissue removal and probe release as described^47^. Tissue removal took place in two separate steps with RLT buffer with β-mercaptoethanol and Proteinase K. 80 µl of 1% β-mercaptoethanol (Sigma-Aldrich, USA) in RLT buffer (Qiagen, Germany) were added to the wells and incubated at 56°C for 1 h. Following removal of the reaction mix and wash with 0.1X SSC solution, 80 µl of second tissue removal mixture; 2.5 µg/µl Proteinase K in PDK buffer (Qiagen, Germany) were added and the reaction was performed at 56 °C for 1 h. The complete reaction mix was again removed and a slide wash with one 10 minute wash of the wells with 2X SSC/0.1% SDS (Sigma-Aldrich, USA), followed by 1 min wash with 0.2X SSC and finally 0.1X SSC was performed. Cleavage of probes from the surface was performed in the next steps and not during in situ cDNA synthesis. The reaction mix consisted of 1.1X Second strand buffer (ThermoFisher Scientific, USA), 0.1 mM dNTPs and 1 U/µl USER enzyme (NEB, USA). 75 µl of the mix was added and incubated for 3 h at 37 °C. The released material was collected in a new 96-well PCR plate (Eppendorf, Germany) by aspirating 70 µl of the released material.

### SM-Omics library preparation (2)

Upon initiating the Agilent Bravo form the user was prompted to select either: 1, 2, 3, 4, 6 or 12 columns of the 96-well plate to run. Two positions on the Bravo deck had Peltier thermal stations (4–95 °C) in the standard 96-well format. A reagent plate was prepared for robotic aspiration, transfer and dispensing of reagents. First, single-stranded cDNA was made to double-stranded material using 5 µl of the reaction mix (2.7X First strand buffer, 3.7 U/µl DNA polymerase I and 0.2 U/µl Ribonuclease H (all from ThermoFisher Scientific, USA)) for 2 h at 16°C. Thereafter, the material was blunted by the addition of 5 µl of 3U/µl T4 DNA polymerase (NEB, USA) for 20 min at 16 °C. The reaction was stopped by addition of Invitrogen UltraPure 0.5 M EDTA (pH 8.0, ThermoFisher Scientific, USA) to a final concentration of 20 mM. The material was then purified using Ampure XP (Beckman Coulter, USA) at a bead to cDNA ratio of 1:1. Next, 27.8 µl of the T7 reaction mix (46.2 mM rNTPs, 1.5X T7 reaction buffer, 1.54 U/µl SUPERaseIN inhibitor and 2.3 U/µl T7 enzyme; all from ThermoFisher Scientific, USA) was added and sealed with 40 µl of Vapor-Lock oil (Qiagen, Germany) for an overnight 14 h incubation at 37 °C. After incubation, 2.1 µl of nuclease-free water (ThermoFisher Scientific) was added and the Vapor-Lock was removed, followed by a bead cleanup with RNAclean Ampure XP beads (Beckman Coulter, USA) at a ratio of 1.8:1 of beads:aRNA. The material was then assessed with a Bioanalyzer RNA 6000 Pico kit (Agilent Technologies, USA). 8 µl of the eluted 10 µl aRNA was transferred into a new 96-well PCR plate (Eppendorf, Germany).

### SM-Omics library preparation (3)

2.5 µl of either 3 µM (standard) or 15 µM aRNA adapters (efficient) [rApp]AGATCGGAAGAGCACACGTCTGAACTCCAGTCAC[ddC] were added to 8 µl of aRNA. The reaction was then incubated at 70 °C in a PCR machine for 2 min and immediately chilled on wet ice. The user then again selected the number of columns they wished to run. 4.5 µl T4 RNA ligation mix (3.3X T4 RNA ligase buffer, 66U/µl truncated T4 ligase 2 and 13U/µl murine RNAse inhibitor (all from NEB, USA)) were added to the aRNA/adapter solution. The ligation reaction took place at 25 °C for 1 h (standard) or 3 h (efficient). For the SM-Omics protocol, the ligation reaction was performed for 3 h in the presence of 15 µM aRNA adapters. The ligation was followed by an Ampure XP (Beckam Coulter, USA) bead purification at a ratio of 1.8:1 bead:cDNA. Elution volume was 12 µl. After bead purification, 2 µl of a primer and dNTP mix (1:1 v/v of either 20 µM or 40 µM GTGACTGGAGTTCAGACGTGTGCTCTTCCGA and 10 mM dNTPs) were added to the ligated samples. For the SM-Omics protocol, 40 µM primer amount was added using the same volumes. Then, the samples were sealed with 40 µl Vapor-Lock (Qiagen, Germany) and heated to 65 °C for 5 min. The Vapor-Lock was thereafter removed and 8 µl of reverse transcription mix were added (2.5X First strand buffer, 13 mM DTT, 5 U/µl RNaseOUT and 25 U/µl Superscript III; all from Thermo Fisher Scientific, USA), with the addition of 40 µl Vapor-Lock to reseal the reaction. The samples were incubated at 50 °C for 1 h. 10 µl of nuclease-free water was added followed by a final Ampure XP bead purification at 1.7:1 bead:cDNA ratio with a final elution of 10 µl nuclease-free water.

### Staining tissues with oligonucleotide-conjugated antibodies

As described above, the fresh frozen tissue was placed on the spatial array slide and fixed at RT, followed by antibody incubations at 4 °C. First, tissues were blocked and permeabilized as described above. This was followed by a series of 3 washes in 1X PBS and a last wash that was incubated for 2 min. After discarding the wash, oligonucleotide-conjugated antibodies and fluorescently labeled antibodies (Biolegend, USA) were both added at a 1:100 dilution in the same buffer as in the initial permeabilization step and incubated for 1 h. The tissue was then washed and the antibody conjugates fixed to the array surface in 4% PFA (Sigma-Aldrich, USA). Tissues were then fluorescently imaged and SM-Omics libraries created. The following steps were added in the library preparations to ensure collection of spatially DNA-barcoded antibody tags. First, cDNA synthesis was performed in situ under the same conditions as described above. Next, second strand synthesis was also performed as described followed by an Ampure XP bead clean up as according to manufacturer’s instructions. During this clean up, material that would otherwise have been discarded after binding to the beads in standard SM-Omics library preparations, was saved and represented a population of spatially DNA-barcoded antibody tags. This elute contained short products that required a bead clean up procedure as well, where a 1.4X bead-to-material ratio was used and the final product eluted in 50 µL EB (Qiagen, Germany). This material was then indexed for Illumina sequencing using Small RNA Illumina indexes in a KAPA indexing reaction as described in *Quantification, indexing and sequencing*.

### Manual ST library preparation

Manual library preparation was performed as described in Salmén et al.^47^ and included the same experimental steps as the robotic library preparation protocol, but performed manually, incubations took place in a PCR System Eppendorf Mastercycler (Eppendorf, Germany) and instead of Vapor-Lock, reactions were sealed using MicroAmp Optical 8-Cap Strips (ThermoFisher Scientific, USA). The manual procedure also included the following deviations from the robotic library preparation: T7 reaction mix of 18.6 µl was used and 1.4 µl of nuclease-free water was added after the 14 h incubation.

### Manual visium preparation

Cortical tissues from an adult mouse brain were cryosectioned at 10 µm thickness and placed on Visium capture areas. The protocol was followed as in the Visium Spatial Gene Expression User Guide CG000239 Rev B as provided by 10X Genomics.

### Quantification, indexing and sequencing

qPCR library quantification and indexing were performed as described in Salmén et al.^47^. The indexed SM-Omics cDNA libraries were diluted with 40 µl of nuclease-free water to allow for a final library bead cleanup with 0.8:1 ratio Ampure XP beads to PCR products, according to the manufacturer’s protocol. Final elution was done in 16 µl EB (Qiagen, Germany). Individual libraries’ fragment lengths and concentrations were evaluated on a Bioanalyzer HS (Agilent Technologies, USA) or DNA1000 Tapestation (Agilent Technologies, USA) and DNA HS Qubit assays (ThermoFisher Scientific, USA), respectively. Samples were then diluted to the desired concentration for sequencing (~1.08 pM final for NextSeq sequencing with 10% PhiX) and sequenced 27–30nt in the forward read and 55–58nt in the reverse read. For antibody tags, the final clean-up was performed at 0.9:1 ratio of beads to PCR products and elution again done in 16 µl EB (Qiagen, Germany). Samples were diluted to 8pM final concentration before sequencing on an Illumina Miseq (2 × 25nt).

### Statistics and reproducibility

#### Raw reads processing and mapping

ST, SM-Omics, Visium or antibody tag fastq reads were generated with bcl2fastq2. ST Pipeline^59^ v.1.7.6 was used to demultiplex the spatial barcodes and collapse duplicate UMI sequences for ST, SM-Omics and Visium. In short, 5nt trimmed R2 was used for mapping to the mouse genome (GRCm38 primary assembly available at https://www.ncbi.nlm.nih.gov/assembly/GCF_000001635.20/) using STAR (v2.6.0)^60^. After that, mapped reads were annotated using HTseq-count (v0.11.4)^61^ using the m11 gtf file (https://www.gencodegenes.org/mouse/release_M11.html). To collapse UMIs, the annotated reads needed to first be connected to a spatial barcode using a TagGD^59,62^ (v0.3.6) demultiplexer (k-mer 6, mismatches 2). Then, UMIs mapping to the same transcript and spatial barcode were collapsed using naive clustering with one mismatch allowed in the mapping process. The output file was a genes-by-barcode matrix that was used in all further processing steps. To map antibody tags to their respective spatial barcodes, we used the tag quantification pipeline originally developed for CITE-Seq (v.1.4.3) available at https://github.com/Hoohm/CITE-seq-Count. The pipeline was run with default parameters (maximum Hamming distance of 1). We additionally provided the spatial barcodes and corrected the spatial mapping (1 mismatch) for a total of 1007 different barcodes.

#### SpoTteR: automated image registration for spatial transcriptomics arrays

For efficient processing, HE images were scaled to approximately 500 × 500 pixels using the imagemagick (https://imagemagick.org/index.php) mogrify command. Other image operations as mentioned below were performed using the R package imager (http://dahtah.github.io/imager/) unless specified differently. In order to reconstruct the positions of all ST spots, visible (*i.e*., not covered by the tissue section) barcode (x,y) spots were registered through blob detection and then refined by keeping only those blobs (i.e. potential grid points) that were likely to be part of a regular grid. Blob detection refers to finding circular features of a predefined size in the image. To prepare the H&E image for blob detection, the tissue section was masked generously through 10% quantile thresholding in a user-defined color channel as obtained through the function imsplit. The borders of the resulting image were cropped four pixels from each of the four image borders in order to remove any abnormality or border effects that might interfere with blob detection. For blob detection, we first blurred the cropped image isotropically using the function isoblur with sigma = 3 and then computed the image hessian (function imhessian). This allowed us to detect probable blob centers using the function dilate_square with size = 3 and the function pad with nPix = 4 and pos = −1. Blob centers (i.e., potential grid points) that were likely part of a regular grid structure were selected by calculating the x and y distances between all detected blob centers. Those blob centers that based on their 8 nearest neighbors had a high (empirically determined) combined grid score (metric based on distance and grid angle between the neighboring centers) were kept. A regular grid was then fitted to these potential grid points using a custom optimizer built around the function nlminb of the R package stats, that minimizes the distance of potential grid points to the suggested regular grid, while assuming 90° angles and 42 grid points per row and column. This first, rough grid initialized an iterative process in which the 0.1% potential grid points that least fit the grid were removed in each iteration, the number of grid points per row and column were updated accordingly, and a new grid was fitted until the target number of grid points per row (here 35) and column (here 33) was reached. The grid design and target number of rows and columns is fully adjustable in SpoTteR. Finally, those grid points that overlapped the tissue sections were identified by building a mask that represented the tissue area and registering all grid points that were present in this mask. To build this mask, we calculated the mean and standard deviation of the background intensity based on the first 20 pixels from the image border, because no tissues were expected in that area. Pixels with an intensity greater than the mean background intensity adjusted for its standard deviation were set to 1 (likely tissue) and those below or equal the mean background intensity adjusted for its standard deviation were set to 0 (likely background), creating the primary tissue mask. Complementarily, a background mask was created by selecting all likely background pixels using the function px.flood with sigma = 0.1 and removing those pixels from the primary tissue mask in order to remove dark artefacts. Two final rounds of isotropic blurring using the function isoblur with sigma = 10 and sigma = 1 in combination with intensity thresholding enhanced the detection of weakly colored tissue regions such areas around the tissue edges. In order to further accommodate atypical tissue coloring, bubbles, and smears present as imaging artifacts, we introduced a parameter to specify the usage of the green color channel instead of the red color channel for tissue detection, which exploits the observation that smears and H&E staining artefacts often lead to spurious pink coloring, which is especially strong in the red channel. To address bubbles, another common image artefact, we introduced a parameter that allows the creation of an additional bubble mask based on all three color channels that specifically identifies bubbles as features that have roughly the same low intensity in all three color channels as they are typically dark gray or even black in color. The thus identified likely bubble pixels are then also removed from the tissue mask. These two additional (TRUE/FALSE) parameters enable to easily process data from tissues of various degrees of coloration and bubble artefacts. Finally, an intermediate report notifies the user of irregularities in the automatic alignment process and allows for visual inspection. The output.tsv file contained barcode spots (x,y) as centroid pixel coordinates of the detected grid, as well as a TRUE/FALSE value, set as TRUE if the barcode spot was detected as under the tissue section area.

#### SpoTter integration with ST pipeline and quality control reporting

The following steps integrate the output from the automated image alignment steps with the output gene-by-barcode expression file as produced by the ST Pipeline v.1.7.6. The barcode (x,y) spots approximated as under the tissue section were used for subsetting the ST Pipeline gene-by-barcode file. Then, the original H&E images were downscaled and cropped using the following imagemagick commands: convert HE_image.jpg -crop width“x“height+xa+ya; where width and height represented the Euclidean lengths between (x,y) grid detected barcode spots (33,35), (1,35) and (1,35), respectively. xa and ya were described as the centroid pixel coordinates of the grid point (33,35). The cropped H&E image was then rotated as follows: mogrify -flop -flip HE_image.jpg and this image was then used as input to the QC reporting system and for the GUI annotation tool. A final quality control (QC) report was created when running SpoTteR. All code for running image registration and QC reporting with SpoTteR has been made available at: https://github.com/klarman-cell-observatory/SpoTteR.

#### Comparison of SpoTter *vs*. ST spot detector vs. manual alignment

To be able to compare the automated image processing developed here to that of manually processed images, we acquired an additional image of the ST array area after the experiment was performed and the tissue had been removed from the array surface. Briefly, complementary and Cy3 labeled oligonucleotides (IDT, USA) were diluted in 2X SSC with 0.05% SDS to a final concentration of 1 µM. 50 µl of the diluted solution was added to the array surface and incubated with shaking (50 rpm) for 10 min at RT. This was followed by washing the slide in 4XSCC with 0.1% SDS and 0.2X SSC. The array frame and all ST barcode positions had then efficiently been labeled and acquired on the same imaging system as described. All input images in the following comparisons were the same approximate input sizes and resolution. The ST spot detector tool previously developed^48^ uses the H&E and Cy3 images as input. Due to its intrinsic scaling factor and input image size requirements, initial pre-processing of both images was needed, such that images be linearly downscaled to 30% of their original size and both images individually cropped to represent the same FOVs as collected during the imaging step. However, cropping was only needed if the user did not have the possibility to automatically acquire the same FOVs using the same starting (x,y) positions. For manual alignment, we used Adobe Photoshop for initial pre-processing, same as in the previous step. Both the H&E and Cy3 acquired images were downscaled to 30% of their original size, rotated 180 degrees and aligned to the same starting (x,y) pixel coordinates. This was followed by cropping both images along the middle of the first and last row and column. The tissue boundaries were detected using the magic wand function (32px) and the selection subtracted in the Cy3 image. Spots boundaries were again detected using the same magick wand function and the background noise cleaned up using the bucket fill function (250px) in a grayscale image. This grayscale image was further used in Fiji^63^ to detect the centroid coordinates of each ST barcode spot. Following Fiji processing, we translated (x,y) pixel centroid coordinates to ST barcode spot coordinates (as given during the demultiplexing step in the ST pipeline). For SpoTteR input, we only provided the original H&E imaged as acquired by the imaging system with no GUI-based preprocessing. For speed comparisons, total time needed for preprocessing steps was measured first. For manual processing, the pre-processing steps included alignment of the H&E and Cy3 images with Adobe Photoshop 2019 and creation of an ST array spots files. For ST Detector pre-processing time, we only took into consideration the time needed to open the same images in Adobe Photoshop, downscale them to 30% size and crop them the same size without any other image handling processes performed. For SpoTteR, preprocessing included the downscaling step performed with imagemagick and incorporated into the workflow. Processing steps were then performed and time was measured as described before. Total speed was considered as 1/t [s^−1^] where t represents the sum of time needed for both the pre-processing and processing steps. False positive and negative rates were calculated as percentage of spots present or absent in SpoTteR or ST Detector as compared to manually processed spot coordinates.

#### Estimating lateral diffusion

Two consecutive mouse cortex fresh frozen sections were processed. One was processed manually as described earlier^47^ while the other was processed using our devised robotic liquid handling setup. For these tests, we created poly(d)T arrays in-house according to manufacturer’s instructions (Codelink, Surmodics, USA) using amine-activated slides. The surface area covered with poly(d)T probes was 6x6mm. Both the H&E and gene activity Cy3 images were processed in Fiji^63^. First, in order to detect the nuclear boundaries of cells chosen at random throughout the tissue, we drew a line (Straight > Freehand line) through each visible nucleus (*n* = 50). Secondly, we collected pixel intensities and distances reaching through each of the chosen nuclei and its surrounding area (Analyze > Plot Profile). To distinguish nuclear boundaries in the collected intensity *vs*. distance data, we first fit a 5th degree polynomial of the curve. Then, we found local minima and maxima in each curve and determined cell boundaries as local minima present at above 10% signal intensity of the local maximum value for each curve. After cell boundaries were defined, we repeated the process using the Cy3 fluorescent gene activity image. Finally, we measured the distance between the detected Cy3 and nuclear signals for each selected cell. Left and right cell boundaries representing opposite sides of each cell were used in the estimate in each condition. A 0.1728 pixel to distance conversion ratio was used to transform pixels to micrometers reported in this paper. If a diffusion distance measure was scored as negative it implied that the Cy3 signal was contained within the detected cell boundaries, and positive if outside those same boundaries.

#### Estimating reproducibility of SM-Omics in situ reactions

Scikit-image^64^ was used to process the H&E and respective fluorescent gene expression images. First, a grayscale fluorescent image was smoothed using a Gaussian filter (sigma = 0.01). Then, we applied morphological reconstruction by dilating the image edges through filtering its regional maxima. This enabled us to create a background image value that could be subtracted from the original image and used in further analysis. Then, we created an elevation map with a Sobel filter to mask the elevated points. This image could then be used in a tissue (i.e., object) detection step using watershedding. The inverted tissue boundaries were subtracted from the detected fluorescent tissue gene expression signals and used in all further analysis. The means of the fluorescent signals were compared using a two-sided Wilcoxon’s rank-sum test. If the expected signal-to-noise ratio between the detected gene expression signature and background signals was less than 3:1 new tissue optimizations are recommended.

#### Annotation patterns through manual image annotation and registration

To manually annotate tissue images based on their H&E features, we used a previously adapted graphical and cloud-based user interface^26^. We assigned each ST (x,y) coordinate with one or more regional tags. The region names used to annotate MOB were: granular cell layer (GR), outer plexiform layer (OPL), mitral layer (MI), internal plexiform layer (IPL) and glomerular layer (GL) and to annotate mouse cortex were: cerebral nuclei (CNU), cortical subplate (CTXsp), fiber tracts, hippocampal formation (HIP), hypothalamus (HY), isocortex (ISOCTX), midbrain (MB), piriform area (PIR) and thalamus (TH). For annotating spleen, we used four major areas: red pulp, B-follicle, marginal zone and periarteriolar lymphoid sheaths (PALS). To overlay tissue images through an image registration task, we used centroids of each annotated region as anchor points in the image translation and rotation tasks, as previously described^32^. This allowed us to display the data in a common coordinate system and to highlight genes and annotation areas of interest.

#### Comparisons between spatial gene expression profiles

For comparisons between the SM-Omics and ST datasets, reads were first downsampled to the same saturation level before invoking the ST pipeline mapper, annotator and counter run to receive UMIs per spatial (x,y) barcode. Depending on sequencing depth, a gene was counted as expressed if the corresponding transcript was present in more than 10^−6^ of the sequencing depth. The total count over all spots per gene and sample were then normalized^65^. Spearman’s correlation coefficient between the average and normalized samples was calculated using Scipy v1.2.0^66^. To compare the performance of Visium and SM-Omics, we sequenced both libraries to an average depth of ~65 million paired end reads. For Visium, we sequenced 29 nt in the forward and 43 nt in the reverse read. Reads were downsampled to the same saturation level. Both datasets were processed using the ST pipeline as described above. Conventional GTF files used in the annotation step with HTseq-count were converted so that all transcript features now carried an exon tag used in counting transcripts. UMI collapsing was done using a naive approach and allowing for 1 low quality base present in either of the datasets. Unique molecular identifiers per measurement were calculated as described earlier.

To visualize the counts data per condition, total numbers of detected genes or UMIs were plotted as violin plots and summarized mean values for all replicate libraries overlayed as dotplots; similar as presented in Lord et al.^67^. To compare between different spatial RNA-seq protocol versions, we followed an approach similar to that previously described in Svensson et al.^68^. Raw data were first processed as described in the *Saturation curve generation* section, and each replicate (at least *n* = 3) from each condition (i.e., spatial RNA-seq protocol version) was represented by the counts mean^67^ at each of 9 different saturation points. Following processing, summarized counts data in each comparison were first scaled [0,1] and then used to estimate a generalized linear mixed model (glmm). We used a glmm (R package glmmTMB v1.1.1) modeled as a proportional binomial logit response between counts, protocol version (fixed effect) and replicate (random effect). Log proportions of annotated reads were used as offsets in the model. All glmm estimates were performed using the R stats package (v4.0.1) and Wald’s p-values reported.

#### Saturation curve generation

Number of unique molecules was calculated by subsampling the same proportion of mapped and annotated reads from each sample. First, each library was randomly down-sampled to three sequencing saturation points (defined as percentage of raw reads in a library) and numbers of UMIs or unique genes and annotated reads in a sample collected after running the ST Pipeline v.1.7.6 as described in the *Raw reads processing and mapping* section above. Using this information, we could solve the Lineweaver–Burk equation and accurately estimate the number of raw reads *R* in each sample *s* that are needed to reach a certain saturation level *S* in a given library:

1 $${R}_{s}=\frac{{S}_{s}\times {K}_{M}}{{V}_{{\max }}-{S}_{s}},$$

where *V*_max_ is the maximum saturation point and *K*_*M*_ represents the number of raw reads at half of *V*_max_ After randomly down-sampling all the libraries to the same library saturation, we considered this our maximum saturation point (100%) in all comparisons and sampled a total of 9 different points (0.001–100%) to be included in the saturation curve plots presented in this paper.

#### Quantitative immunofluorescence profiles per SM-Omics spot

First, we trained a random forest classifier using the Ilastik^69^ (v1.3.3) framework to extract probabilities of the positive class assignment ie. positive antibody signals from our IF mouse brain images. Separate classifiers were trained to each antibody used and a total of ~10 images with at least 10 fields of view were used in the training process. In each classifier, we used two labels for classification: signal and background. Respective full-sized fluorescent microscopy images were then processed and output probabilities used in the following steps. For spleen data, raw fluorescent images were used as input in the following steps. First, images were processed as described in *Estimating reproducibility of SM-Omics* in situ *reactions*. Calculated background was removed from each image, signal boundaries estimated using watershedding followed by creating a binary mask image. This mask was then overlaid with the original fluorescent image and this image was then used in all following steps. To quantify the fluorescent signal intensities per ST spot, the image was cropped into a 33 × 35 matrix creating smaller patches; each patch sized at ±1% image from the centroid of each ST spot. Finally, the intensity from each spot area was calculated as the sum of the fluorescent signal detected in that spot patch.

#### Spatial gene and antibody-based expression analysis

Statistical analysis of the spatial gene and antibody tag expression data was performed using Splotch’ one- or two-level hierarchical model as previously described^32^. In short, the model captures spatial expression in anatomical regions while accounting for experimental parameters such as, in our case, different animals, and calculates gene or antibody expression estimates for each single gene or antibody in each annotated spatial spot. To find targets which were differentially expressed in an annotated morphological region, we computed a one-*vs*-all comparison and took those values with a positive log Bayesian factor (BF). Posterior probabilities presented hereinafter normalized expression estimates and were used throughout the analyses presented. For scaling per annotated region, normalized expression values were first grouped by annotated region and then scaled from 0 to 1 within each sample. The correlation between gene expression and fluorescent signal was calculated in the same way, but the fluorescent signal matrix, prepared as explained in *Calculating quantitative immunofluorescence profiles per SM-Omics spot*, was used instead of the antibody tag counts matrix.

### Comparison to Allen Brain Atlas data

To validate our findings, we downloaded in situ hybridization (ISH) gene expression data from the Allen Brain Atlas^50^ (https://mouse.brain-map.org/) with Image Credit: Allen Institute for Brain Science. The following gene expression images were used from the ABA as denoted with appropriate image and experiment identifiers: *CTGF* 478 [https://mouse.brain-map.org/experiment/show/79556634], *CAMK4* 474 [https://mouse.brain-map.org/experiment/show/75038464], *LANCL3* 474 [https://mouse.brain-map.org/experiment/show/73925716], *CBLN4* 476 [https://mouse.brain-map.org/experiment/show/72283804], *NR2F2* 466 and 250 [https://mouse.brain-map.org/experiment/show/112646890], *NRSN1* 478 [https://mouse.brain-map.org/experiment/show/71358557], *NOS1AP* 472 [https://mouse.brain-map.org/experiment/show/77280574], *CDH23* 469 [https://mouse.brain-map.org/experiment/show/72283805], *PRSS12* 474 [https://mouse.brain-map.org/experiment/show/71836879], *CABP7* 253 [https://mouse.brain-map.org/experiment/show/73930835], *SEMA4G* 266 [https://mouse.brain-map.org/experiment/show/71587856], *DKKL1* 237 [https://mouse.brain-map.org/experiment/show/70634395], *SLC17A6* 272 [https://mouse.brain-map.org/experiment/show/73818754] and *PENK* 262 [https://mouse.brain-map.org/experiment/show/74881286]. For comparisons in MOB samples, we used the following regions from ABA: GL, GR, MI and OPL. For comparison in cortex samples, we used the following regions from ABA: piriform-amygdalar area (PAA), postpiriform transition area (TR) in addition to CNU, CTXsp, HIP, HY, ISOCTX, MB and TH. Prior to enrichment analysis, genes found in PAA, TR and PIR in ABA were merged into one region name: PIR. We filtered genes with fold change >1 and expression threshold >2.5 in ABA and compared to genes with positive fold change and log(BF) in our Splotch data and computed a one-sided Fisher’s exact test using Scipy v1.2.066. FDR was estimated using the Benjamini-Hochberg^70^ procedure. Heatmaps denoting regions present in both conditions were plotted. One of the top most differentially expressed genes in both SM-Omics and ABA was chosen from each region and its expression visualized. A reference ST dataset^24^ was also analyzed using Splotch with the same settings as used for SM-Omics, visualized and compared to SM-Omics. To create correlations between ABA expression patterns and SM-Omics, Visium and ST expression patterns, normalized expression data was first grouped by annotated region and then scaled from 0 to 1 within each sample. To compare SM-Omics and ST, we compared top genes per MOB region: *NRSN1*, *NOS1AP*, *CDH23* and *PRSS12*. To compare SM-Omics and Visium, we compared top genes per mouse brain cortex as found in ABA: *ADORA2A*, *CABP7*, *SLC6A11*, *IER5*, *SLC17A6* and *GREM2*.

### Reporting summary

Further information on research design is available in the Nature Research Reporting Summary linked to this article.

## Supplementary information

Supplementary Information

Reporting Summary

Description of Additional Supplementary Files

Supplementary Data 1

SupplementaryData 2

Supplementary Data 3

## Data Availability

Raw sequencing data is available at NCBI’s Bioproject under accession PRJNA797464. All processed and source data generated in this study have been deposited in the Single Cell Portal under accession code SCP979. All other relevant data supporting the key findings of this study are available within the article and its Supplementary Information files.

## Code availability

All code is on GitHub at https://github.com/klarman-cell-observatory/sm-omics.

## References

[1] Jovanovic M (2015). Immunogenetics. Dynamic profiling of the protein life cycle in response to pathogens. Science.

[2] Rabani M (2014). High-resolution sequencing and modeling identifies distinct dynamic RNA regulatory strategies. Cell.

[3] Chick JM (2016). Defining the consequences of genetic variation on a proteome-wide scale. Nature.

[4] Zhang B (2014). Proteogenomic characterization of human colon and rectal cancer. Nature.

[5] Stoeckius M (2017). Simultaneous epitope and transcriptome measurement in single cells. Nat. Methods.

[6] Zheng GXY (2017). Massively parallel digital transcriptional profiling of single cells. Nat. Commun..

[7] Macosko EZ (2015). Highly parallel genome-wide expression profiling of individual cells using nanoliter droplets. Cell.

[8] Klein AM (2015). Droplet barcoding for single-cell transcriptomics applied to embryonic stem cells. Cell.

[9] Habib N (2016). Div-Seq: Single-nucleus RNA-Seq reveals dynamics of rare adult newborn neurons. Science.

[10] Habib N (2017). Massively parallel single-nucleus RNA-seq with DroNc-seq. Nat. Methods.

[11] Slyper M (2020). A single-cell and single-nucleus RNA-Seq toolbox for fresh and frozen human tumors. Nat. Med..

[12] Darmanis S (2016). Simultaneous multiplexed measurement of RNA and proteins in single cells. Cell Rep..

[13] Hanahan D, Coussens LM (2012). Accessories to the crime: functions of cells recruited to the tumor microenvironment. Cancer Cell.

[14] Bodenmiller B (2016). Multiplexed epitope-based tissue imaging for discovery and healthcare applications. Cell Syst..

[15] Chen KH, Boettiger AN, Moffitt JR, Wang S, Zhuang X (2015). RNA imaging. Spatially resolved, highly multiplexed RNA profiling in single cells. Science.

[16] Lubeck E, Coskun AF, Zhiyentayev T, Ahmad M, Cai L (2014). Single-cell in situ RNA profiling by sequential hybridization. Nat. Methods.

[17] Eng C-HL (2019). Transcriptome-scale super-resolved imaging in tissues by RNA seqFISH. Nature.

[18] Lee JH (2014). Highly multiplexed subcellular RNA sequencing in situ. Science.

[19] Goltsev Y (2018). Deep profiling of mouse splenic architecture with CODEX multiplexed imaging. Cell.

[20] Keren L (2018). A structured tumor-immune microenvironment in triple negative breast cancer revealed by multiplexed ion beam imaging. Cell.

[21] Codeluppi S (2018). Spatial organization of the somatosensory cortex revealed by osmFISH. Nat. Methods.

[22] Moffitt JR (2018). Molecular, spatial, and functional single-cell profiling of the hypothalamic preoptic region. Science.

[23] Ke R (2013). In situ sequencing for RNA analysis in preserved tissue and cells. Nat. Methods.

[24] Ståhl PL (2016). Visualization and analysis of gene expression in tissue sections by spatial transcriptomics. Science.

[25] Rodriques SG (2019). Slide-seq: a scalable technology for measuring genome-wide expression at high spatial resolution. Science.

[26] Vickovic S (2019). High-definition spatial transcriptomics for in situ tissue profiling. Nat. Methods.

[27] Wang X (2018). Three-dimensional intact-tissue sequencing of single-cell transcriptional states. Science.

[28] Maynard KR (2021). Transcriptome-scale spatial gene expression in the human dorsolateral prefrontal cortex. Nat. Neurosci..

[29] Thrane K, Eriksson H, Maaskola J, Hansson J, Lundeberg J (2018). Spatially resolved transcriptomics enables dissection of genetic heterogeneity in stage III cutaneous malignant melanoma. Cancer Res.

[30] Asp M (2017). Spatial detection of fetal marker genes expressed at low level in adult human heart tissue. Sci. Rep..

[31] Asp M (2019). A spatiotemporal organ-wide gene expression and cell atlas of the developing human heart. Cell.

[32] Maniatis S (2019). Spatiotemporal dynamics of molecular pathology in amyotrophic lateral sclerosis. Science.

[33] Berglund E (2018). Spatial maps of prostate cancer transcriptomes reveal an unexplored landscape of heterogeneity. Nat. Commun..

[34] Berglund E (2020). Automation of spatial transcriptomics library preparation to enable rapid and robust insights into spatial organization of tissues. BMC Genomics.

[35] Jemt A (2016). An automated approach to prepare tissue-derived spatially barcoded RNA-sequencing libraries. Sci. Rep..

[36] Vickovic S (2016). Massive and parallel expression profiling using microarrayed single-cell sequencing. Nat. Commun..

[37] Lein E, Borm LE, Linnarsson S (2017). The promise of spatial transcriptomics for neuroscience in the era of molecular cell typing. Science.

[38] Gerdes MJ (2013). Highly multiplexed single-cell analysis of formalin-fixed, paraffin-embedded cancer tissue. Proc. Natl Acad. Sci. USA.

[39] Lin J-R, Fallahi-Sichani M, Sorger PK (2015). Highly multiplexed imaging of single cells using a high-throughput cyclic immunofluorescence method. Nat. Commun..

[40] Angelo M (2014). Multiplexed ion beam imaging of human breast tumors. Nat. Med..

[41] Giesen C (2014). Highly multiplexed imaging of tumor tissues with subcellular resolution by mass cytometry. Nat. Methods.

[42] Merritt CR (2020). Multiplex digital spatial profiling of proteins and RNA in fixed tissue. Nat. Biotechnol..

[43] Schulz D (2018). Simultaneous multiplexed imaging of mRNA and proteins with subcellular resolution in breast cancer tissue samples by mass cytometry. Cell Syst..

[44] 10x Genomics. https://www.10xgenomics.com/products/spatial-proteomics.

[45] Fisher S (2011). A scalable, fully automated process for construction of sequence-ready human exome targeted capture libraries. Genome Biol..

[46] Rohland N, Reich D (2012). Cost-effective, high-throughput DNA sequencing libraries for multiplexed target capture. Genome Res.

[47] Salmén F (2018). Barcoded solid-phase RNA capture for spatial transcriptomics profiling in mammalian tissue sections. Nat. Protoc..

[48] Wong K, Navarro JF, Bergenstråhle L, Ståhl PL, Lundeberg J (2018). ST Spot Detector: a web-based application for automatic spot and tissue detection for spatial Transcriptomics image datasets. Bioinformatics.

[49] Äijö, T. et al. Splotch: Robust estimation of aligned spatial temporal gene expression data. 10.1101/757096.

[50] Lein ES (2007). Genome-wide atlas of gene expression in the adult mouse brain. Nature.

[51] Kang H (2001). An important role of neural activity-dependent CaMKIV signaling in the consolidation of long-term memory. Cell.

[52] Mann AP (2017). Identification of a peptide recognizing cerebrovascular changes in mouse models of Alzheimer’s disease. Nat. Commun.

[53] TotalSeq^TM^. https://www.biolegend.com/en-us/totalseq.

[54] Nagayama S, Homma R, Imamura F (2014). Neuronal organization of olfactory bulb circuits. Front. Neural Circuits.

[55] Zeisel A (2018). Molecular architecture of the mouse nervous system. Cell.

[56] Stickels RR (2021). Highly sensitive spatial transcriptomics at near-cellular resolution with Slide-seqV2. Nat. Biotechnol..

[57] Chen, A. et al. Large field of view-spatially resolved transcriptomics at nanoscale resolution. Preprint at 10.1101/2021.01.17.427004 (2021).

[58] Su G (2021). Spatial multi-omics sequencing for fixed tissue via DBiT-seq. STAR Protoc..

[59] Navarro JF, Sjöstrand J, Salmén F, Lundeberg J, Ståhl PL (2017). ST Pipeline: an automated pipeline for spatial mapping of unique transcripts. Bioinformatics.

[60] Dobin A (2013). STAR: ultrafast universal RNA-seq aligner. Bioinformatics.

[61] Anders S, Pyl PT, Huber W (2015). HTSeq-a Python framework to work with high-throughput sequencing data. Bioinformatics.

[62] Costea PI, Lundeberg J, Akan P (2013). TagGD: fast and accurate software for DNA Tag generation and demultiplexing. PLoS ONE.

[63] Schindelin J (2012). Fiji: an open-source platform for biological-image analysis. Nat. Methods.

[64] van der Walt S (2014). scikit-image: image processing in Python. PeerJ.

[65] Svensson V, Teichmann SA, Stegle O (2018). SpatialDE: identification of spatially variable genes. Nat. Methods.

[66] Jones, E., Peterson, P. & Oliphant, T. SciPy: Open Source Scientific Tools for Python. *Scipy* http://www.scipy.org/ (2001).

[67] Lord SJ, Velle KB, Mullins RD, Fritz-Laylin LK (2020). SuperPlots: communicating reproducibility and variability in cell biology. J. Cell Biol.

[68] Svensson V (2017). Power analysis of single-cell RNA-sequencing experiments. Nat. Methods.

[69] Berg S (2019). ilastik: interactive machine learning for (bio)image analysis. Nat. Methods.

[70] Benjamini Y, Hochberg Y (1995). Controlling the false discovery rate: a practical and powerful approach to multiple testing. J. R. Stat. Soc. Ser. B.

