# Supplementary material for: SM-Omics is an automated platform for high-throughput spatial multi-omics
Source: Nat Commun. 2022 Feb 10;13:795. doi: 10.1038/s41467-022-28445-y (PMC8831571; doi:10.1038/s41467-022-28445-y)
Supplement: Supplementary file 1 — Supplementary Information [file 41467_2022_28445_MOESM1_ESM.pdf]

Supplementary Information for:
SM-Omics is an automated platform for high-throughput spatial multi-omics

Vickovic S[1,2,3,4,#,*], Lötstedt B[1,5,6,#], Klughammer J[1], Mages S[1], Segerstolpe Å[1], Rozenblatt-Rosen O[1,8], Regev A[1,7,8,*]

[1]Klarman Cell Observatory Broad Institute of MIT and Harvard, Cambridge, MA, USA.
[2]Department of Biology, Massachusetts Institute of Technology, Cambridge, MA, USA.
[3]New York Genome Center, New York, NY, USA.
[4]Science for Life Laboratory, Department of Biochemistry and Biophysics, Stockholm University, Solna, Sweden.
[5]Science for Life Laboratory, Department of Gene Technology, KTH Royal Institute of Technology, Stockholm, Sweden.
[6]Department of Biological Engineering, Massachusetts Institute of Technology, Cambridge, MA, USA.
[7]Howard Hughes Medical Institute and Koch Institute for Integrative Cancer Research, Department of Biology, Massachusetts Institute of Technology, Cambridge, MA, USA.
[8]Current address: Genentech, 1 DNA Way, South San Francisco, CA, USA.

# These authors contributed equally to this work

* To whom correspondence should be addressed: vickovic@broadinstitute.org (S.V.), aviv.regev.sc@gmail.com (A.R.)

Supplementary Figures

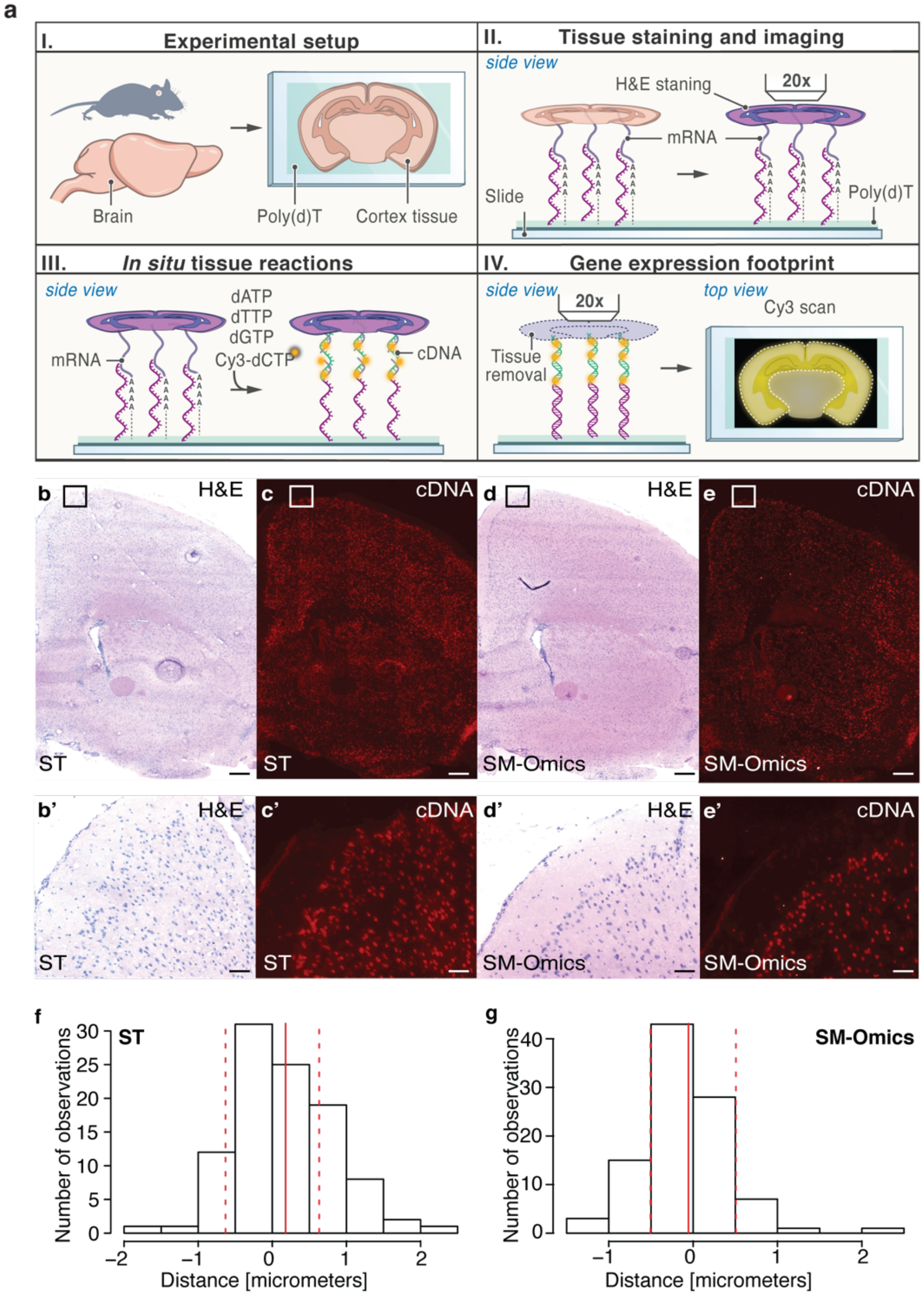

Supplementary Fig. 1. Feasibility of SM-Omics *in situ* reactions. (a) Overview of experimental setup. SM-Omics approach combines automated imaging of H&E (or IF) stained tissue sections to create spatially resolved cDNA expression footprints. First, brain sections are deposited on a mock array with poly(d)T capture area (I) and stained for H&E histology (II). Then, mRNAs are captured on the mock slide and cDNA molecules *in situ* fluorescently labeled (III) to create a spatial cDNA gene expression footprint (IV). **(b)** and **(b')** H&E images of the cortex region on the adult mouse brain for manually prepared ST samples (n=3); coronal brain half (scale bar; 400µm) and zoomed in region (scale bar; 100µm) respectively. **(c)** and **(c')** Fluorescent gene activity cDNA footprints (n=3) corresponding to **(b)** and **(b')**. **(d)** and **(d')** H&E image of the adjacent cortex

region processed with SM-Omics (n=3); coronal brain half (scale bar; 400µm) and zoomed in region (scale bar; 100µm) respectively. **(e)** and **(e')** Fluorescent gene activity footprints (n=3) corresponding to **(d)** and **(d')**. **(f-g)** Histograms of lateral diffusion metrics for ST (**f**) and SM-Omics (**g**). Each bar represents the distance between the detected H&E cell boundary and the detected signal spread for the corresponding gene activity footprint for the same cell (n=50 cells). Solid red lines represent mean and dashed lines standard deviations of the distributions.

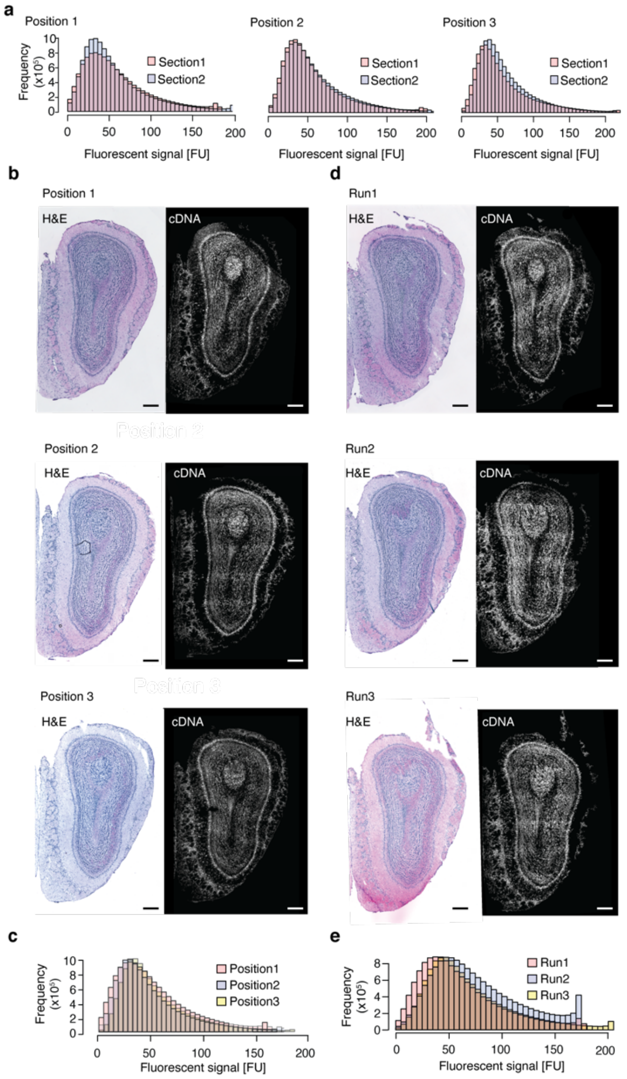

Supplementary Fig. 2. Evaluation of automated *in situ* reactions within and between SM-Omics runs on MOB tissues. **(a)** Histograms of replicate fluorescent tissue footprints (cDNA) detected (**Methods**) in one SM-Omics run and slide position. No significant differences were detected between the means of the distributions (two-sided Wilcoxon's rank-sum test, p-value=0.70). **(b)** H&E images followed by detected fluorescent (cDNA) footprints (**Methods**) reflecting gene activity in the tissue sample (n=2). Each image combination (H&E and cDNA) denotes a respective position (1-3) used during one SM-Omics *in situ* optimization run. Scale bars; 400µm. **(c)** Histograms of fluorescent tissue footprints detected in one SM-Omics run using three slide positions. No significant differences were detected between the means of the distributions (two-sided Wilcoxon's rank-sum test, p-value=0.47). **(d)** H&E images followed by detected fluorescent (cDNA) footprints (**Methods**) reflecting gene activity in the tissue sample (n=3). Each image combination represents a result from a separate SM-Omics run (1-3). Scale bars; 400µm. **(e)** Histograms of fluorescent tissue footprints detected between three SM-Omics runs. No significant differences were detected between the means of the distributions (two-sided Wilcoxon's rank-sum test, p-value=0.24).

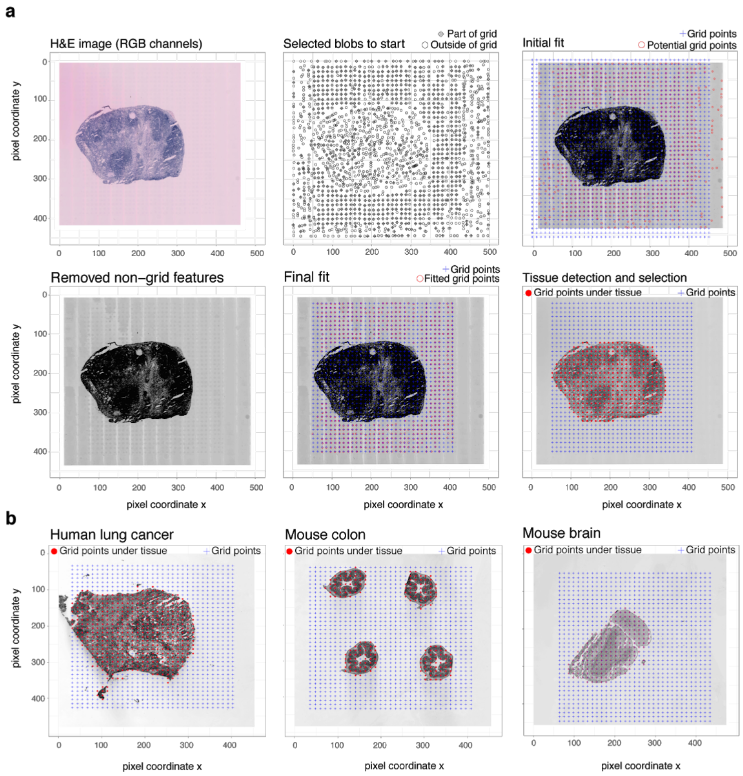

Supplementary Fig. 3. Tissue and array grid detection with SpoTteR. (a) The RGB tissue H&E stained image as input. The RGB image is split into 3 color channels and circular features are detected. Those features that fit a grid pattern (33x35 matrix) are used for the initial fit. Then circular features outside the grid are removed and the process of grid fitting repeated until a perfect 33x35 matrix is adjusted and positioned. Then the tissue is detected and grid spots under the tissue are easily subtracted. **(b)** SpoTteR performance for tissue and grid detection in three different tissue types: human lung cancer, mouse colon and mouse brain.

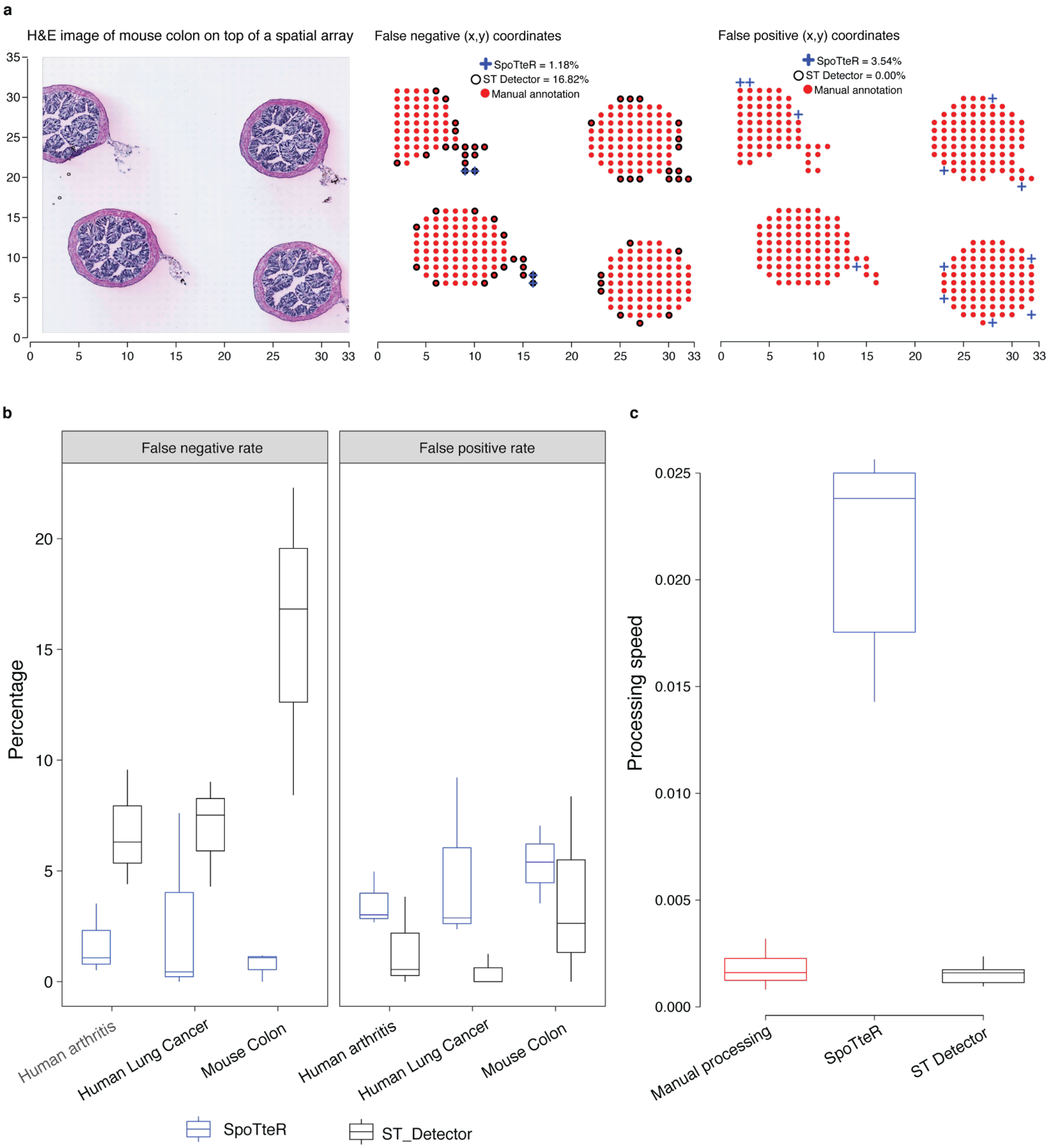

Supplementary Fig. 4. SpoTteR performance. (a) H&E image with corresponding false negative and positive ST barcode spot (x,y) positions using SpoTteR (blue cross) or ST Detector (black circle) as compared to the manually curated positions (filled red circle) for a mouse colon sample. **(b)** Total false negative and positive rates per processed tissue type (n=3) and tested approach (n=3). Center line, median; box, interquartile range; vertical line, 1.5x interquartile range. **(c)** Processing speed (given as 1/time [s^{-1}]) for three tested processing approaches (n=9) with note that there is no hands-on processing needed with SpoTteR while the other approaches require additional user input in either pre-processing or processing steps (**Methods**). Center line, median; box, interquartile range; vertical line, 1.5x interquartile range.

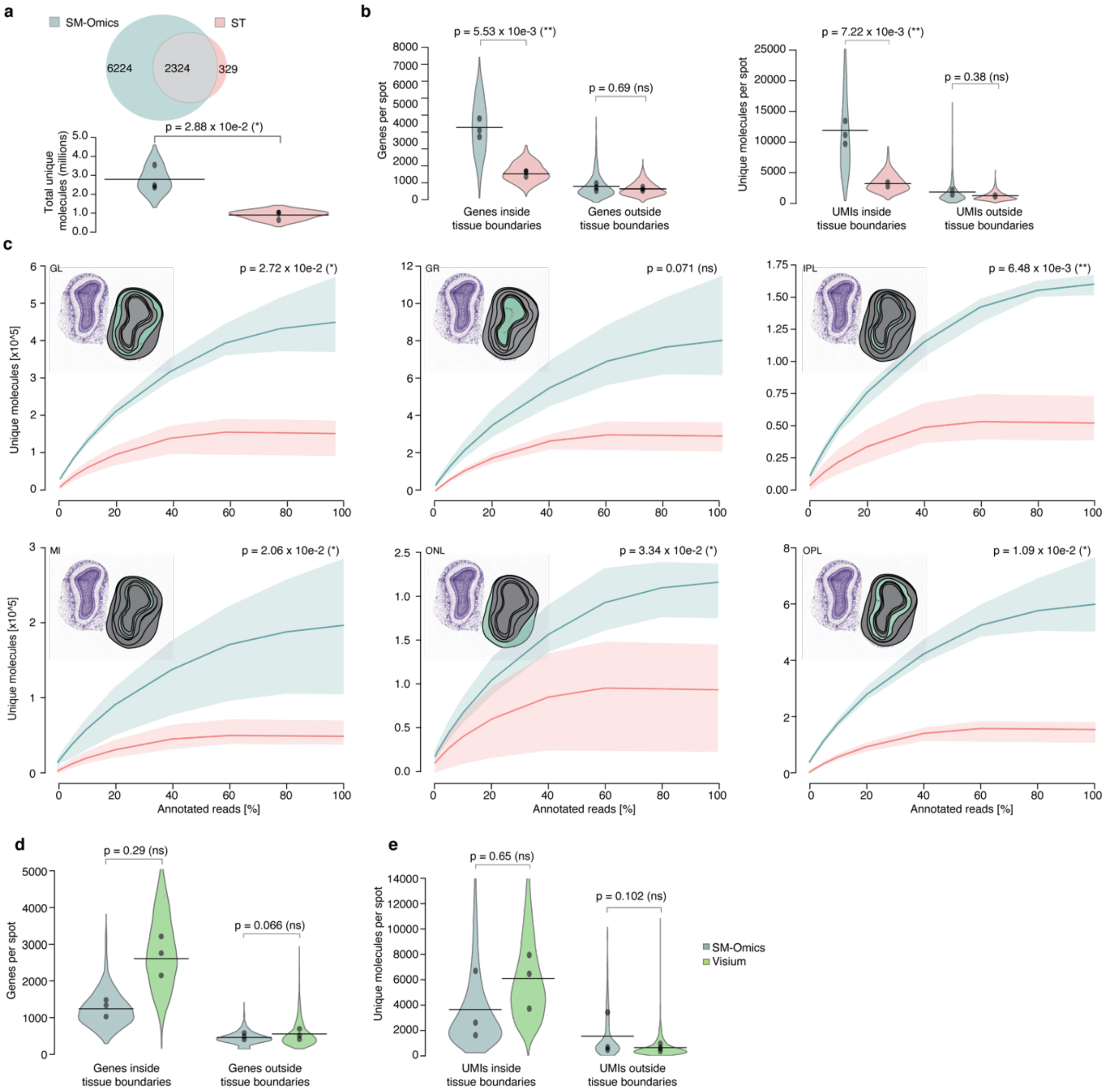

Supplementary Fig. 5. SM-Omics metrics comparisons to other array versions. (a) Comparison of total number of genes and unique molecules in SM-Omics *vs*. ST. Total number of expressed genes and their intersection and total number of unique molecules detected under the tissue boundaries for SM-Omics (blue) and ST (red). Black dots: summarized mean values for replicate libraries: SM-Omics (blue, n=3) and ST (red, n=3) at the highest available sequencing saturation point. Horizontal line: mean of summarized library values (n=3) per condition. **(b)** Comparison of number of genes and unique molecules per spatial spot in SM-Omics *vs*. ST. Total number of expressed genes (left) and unique molecules (right) detected per spatial spot under and outside of the tissue boundaries for all spatial measurements in SM-Omics (blue) and ST (red). Black dots: summarized mean values for replicate libraries: SM-Omics (blue, n=3) and ST (red, n=3) at the highest available sequencing saturation point. Horizontal line denotes the mean of the summarized library values (n=3) per condition. **(c)** Sensitivity of spatial gene expression measurements. Mean number of unique molecules detected per annotated morphological region (y axis) at different library saturation points indicated as proportions of annotated reads (x axis) in

SM-Omics (blue, n=3) and ST (red, n=3). Shaded areas: 95% confidence intervals. Colored line: mean of summarized library values (n=3) per condition. Nissl stain and corresponding annotation regions shown in each subplot where the selected region is shown in green and the rest of the regions in gray. **(d-e)** Comparison of number of genes and unique molecules per spatial spot in SM-Omics *vs*. Visium. Total number of expressed genes **(d)** and unique molecules **(e)** per spatial spot under and outside of the tissue boundaries for all spatial measurements in SM-Omics (blue) and Visium (green). Black dots represent summarized mean values for replicate libraries: SM-Omics (blue, n=3) and Visium (green, n=3) at the highest available sequencing saturation point. Horizontal line denotes the mean of the summarized library values (n=3) per condition. Annotated region abbreviations: GL (glomerular layer), GR (granular cell layer), MI (mitral layer), IPL (internal plexiform layer), ONL (olfactory nerve layer) and OPL (outer plexiform layer). Color legend **(a-c)** is shared between the panels as denoted in (**a**). Color legend **(d-e)** is shared between the panels as denoted in **(e)**. **(a-c)** represents data from adult mouse MOB and **(d-e)** from adult mouse cortex. Statistical significance markings or p-values (Wald's test) are displayed; $0.05<p\leq1$ (ns), $0.01<p\leq0.05$ (*), $0.001<p\leq0.01$ (**). All pairwise comparisons were made at the same respective sequencing depths.

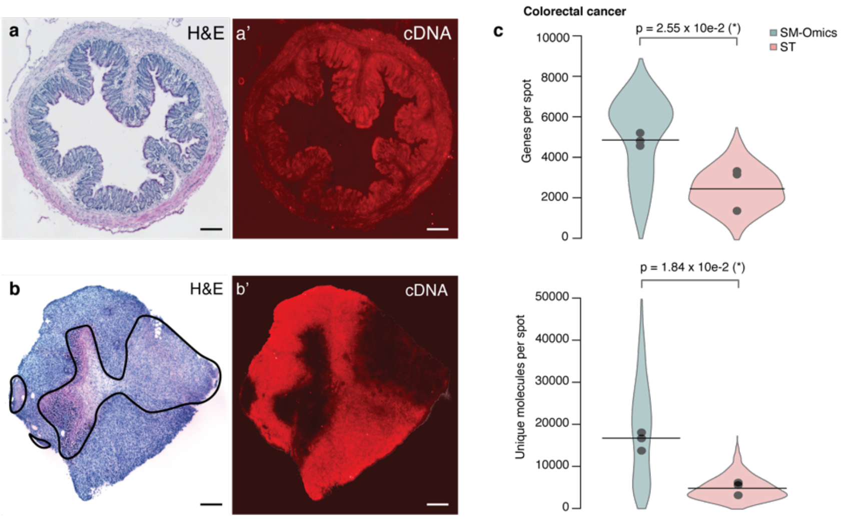

Supplementary Fig. 6. Performing SM-Omics *in situ* reactions on different tissue types.
(a) H&E (left) and **(a')** fluorescent gene activity cDNA footprint (right) for the adult mouse colon (n=3). Scale bas; 200µm. **(b)** H&E (left) and **(b')** fluorescent gene activity cDNA footprint (right) for the preclinical model of colorectal cancer (n=3). Scale bas; 300µm. Dark areas in the cDNA images (**a'** and **b'**) represent areas with no tissue present or areas with extensive tissue necrosis. Bright areas in the cDNA images represent positive cDNA signals. Black lines in (**b)** denote pathologist annotations of necrotic areas. **(c)** Comparison of number of genes and unique molecules per spatial spot in SM-Omics *vs*. ST. Total number of detected genes (top) and unique molecules (bottom) per spatial spot under tissue boundaries for all spatial measurements in SM-Omics (blue) and ST (red) in colorectal cancer. Black dots represent summarized mean values for replicate libraries: SM-Omics (blue, n=3) and ST (red, n=3) at the highest available sequencing saturation point. Horizontal line denotes the mean of the summarized library values (n=3) per condition. Statistical significance p-values (Wald's test) are displayed; $0.01<p\leq0.05$ (*). All pairwise comparisons were made at the same respective sequencing depths.

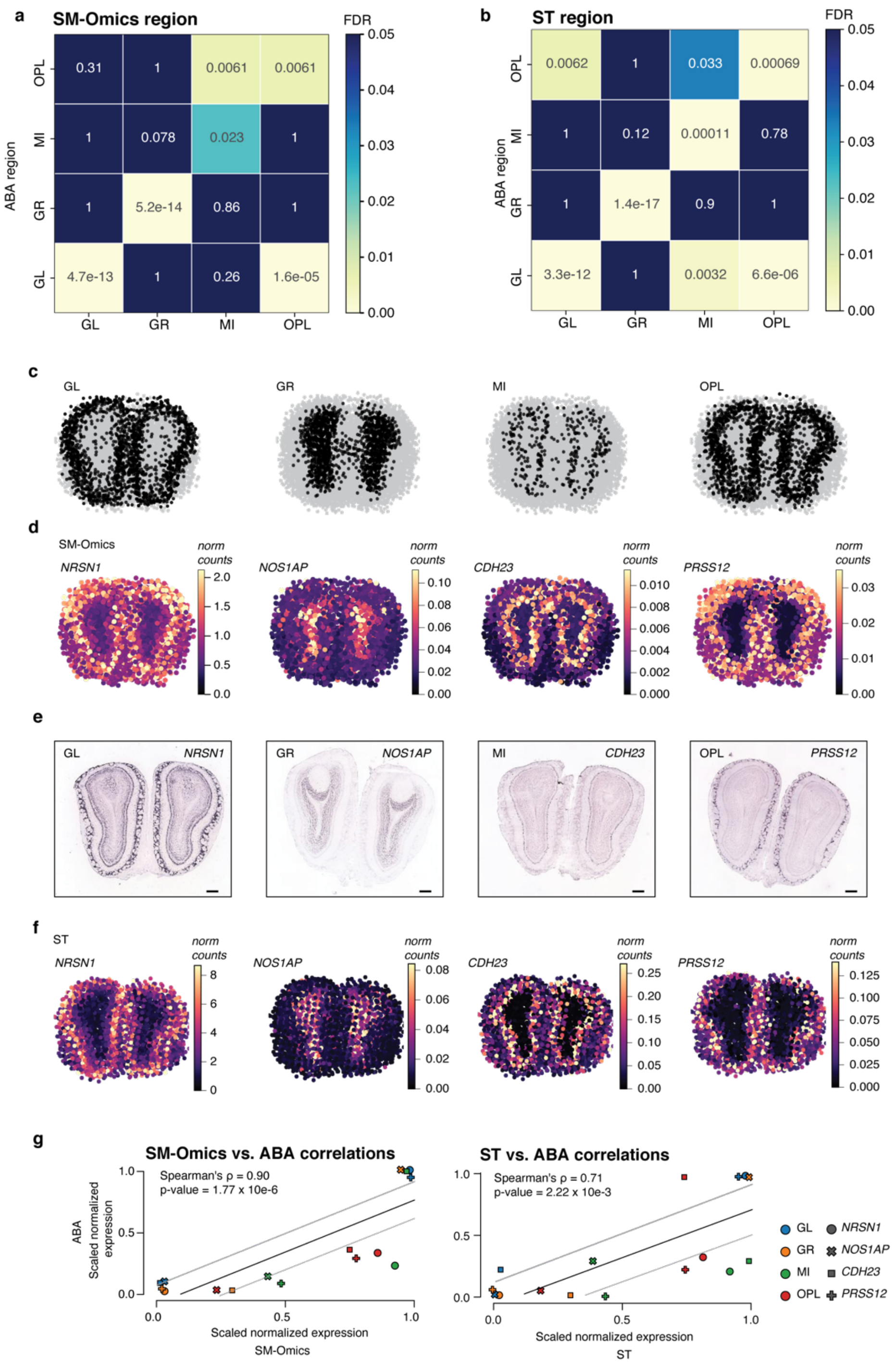

Supplementary Fig. 7. Spatial gene expression specificity, sensitivity and patterns in major annotated layers in SM-Omics and ST in the adult mouse MOB. (a,b) Regional gene expression specificity. Morphological gene expression signatures agree between SM-Omics **(a)**, ST **(b)** and ABA for the major morphological layers. Significance (FDR, Fisher's exact test, one-sided, color scale) for the overlap of genes associated with each layer in SM-Omics **(a)** and ST **(b)** (columns) and in ABA (rows). **(c)** Spatial annotation patterns. Examples of spatial annotation patterns (black) for four major morphological regions (columns) present in the adult MOB

(**Methods**). Spatial points not present in a specific region were marked in gray. **(d-f)** Spatial gene expression. **(d)** Examples of SM-Omics spatial gene expression patterns (normalized expression shown in color scale) for DE genes detected (**Methods**) between the regions GL, GR, MI and OPL with **(e)** corresponding *in situ* hybridization images from ABA (scale bars; 200µm; Image credit: Allen Institute for Brain Science, **Methods**) and **(f)** ST spatial gene expression (normalized expression shown in color scale) for the same genes as in **(d)**. **(g)** Regional gene expression sensitivity. Expression levels (mean scaled signal of all measurements in a region) of each of the same four genes as in **(d)** (spot shape) in each of four regions (spot color) in SM-Omics (x axis, left, scaled normalized expression) or ST (x axis, right, scaled normalized expression) and ABA (y axis, scaled normalized expression). Black line: linear regression with respective standard deviations (gray lines). Annotated region abbreviations: GL (glomerular layer), GR (granular cell layer), MI (mitral layer) and OPL (outer plexiform layer).

a

b

c

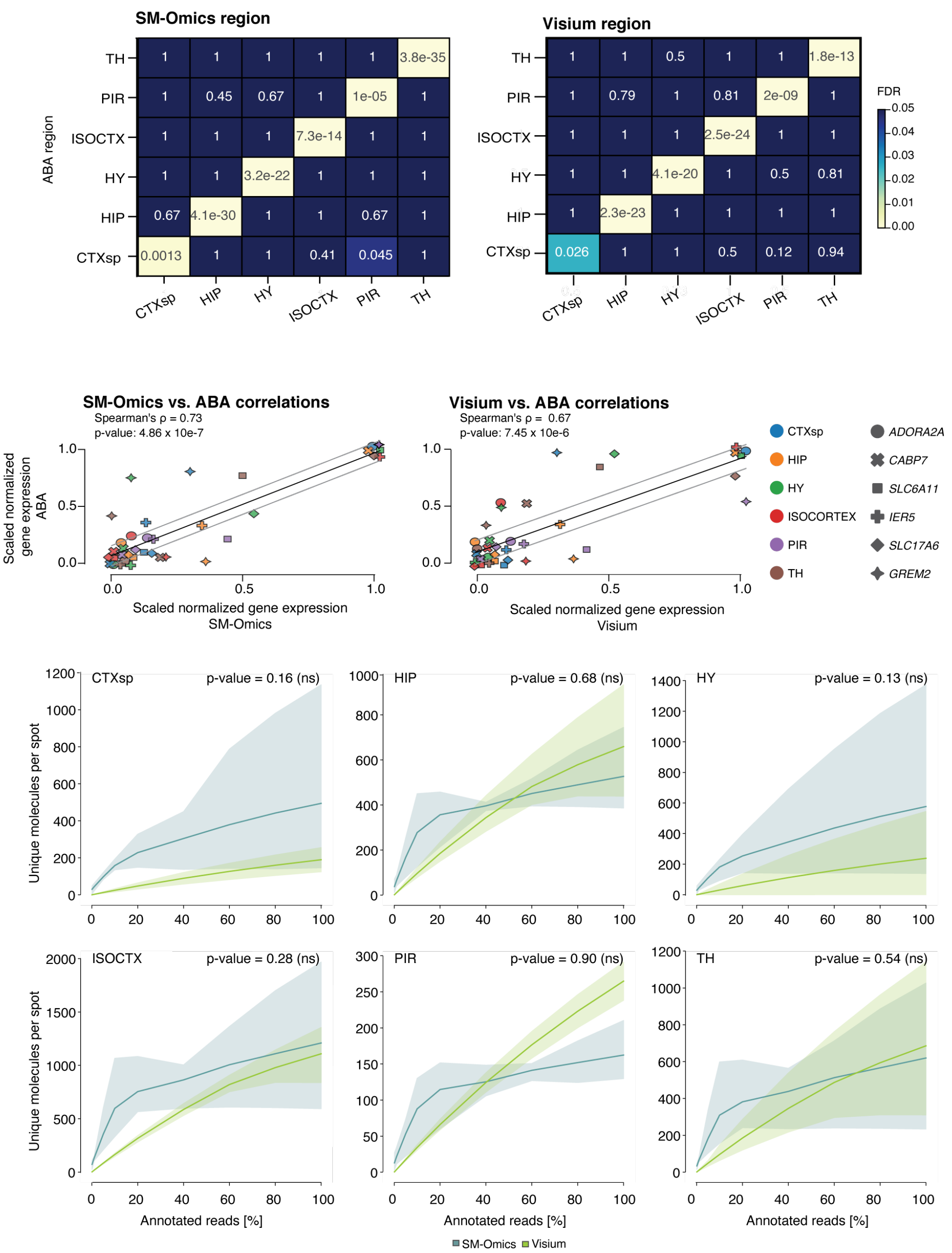

Supplementary Fig. 8. Comparable regional specificity and sensitivity of SM-Omics and Visium in the mouse brain cortex. (a) Morphological gene expression signatures agree between SM-Omics, Visium and ABA for the major cortical layers. Significance (FDR, Fisher's exact test, one-sided, color scale) for the overlap of genes associated with each layer in SM-Omics (left) or Visium (right) (columns) and in ABA (rows). **(b)** Regional gene expression sensitivity. Expression levels (mean scaled signal of all measurements in a region) of each of six genes (spot shape) in each of six regions (spot color) in SM-Omics (x axis, left, scaled normalized expression) or Visium (x axis, right, scaled normalized expression) and ABA (y axis, scaled normalized expression). Black line: linear regression with respective standard deviations (gray lines). **(c)** Method sensitivity

of spatial gene expression measurements. Mean number of unique molecules detected per annotated morphological region (y axis) at different library saturation points indicated as proportions of annotated reads (x axis) in SM-Omics (blue, n=3) and Visium (green, n=3). Shaded areas: 95% confidence intervals. Colored line: mean of summarized library values (n=3) per condition. Statistical significance p-values (Wald's test) are displayed. Annotated region abbreviations: CTXsp (cortical subplate), HY (hypothalamus), HIP (hippocampal formation), ISOCTX (isocortex), PIR (piriform areas) and TH (thalamus).

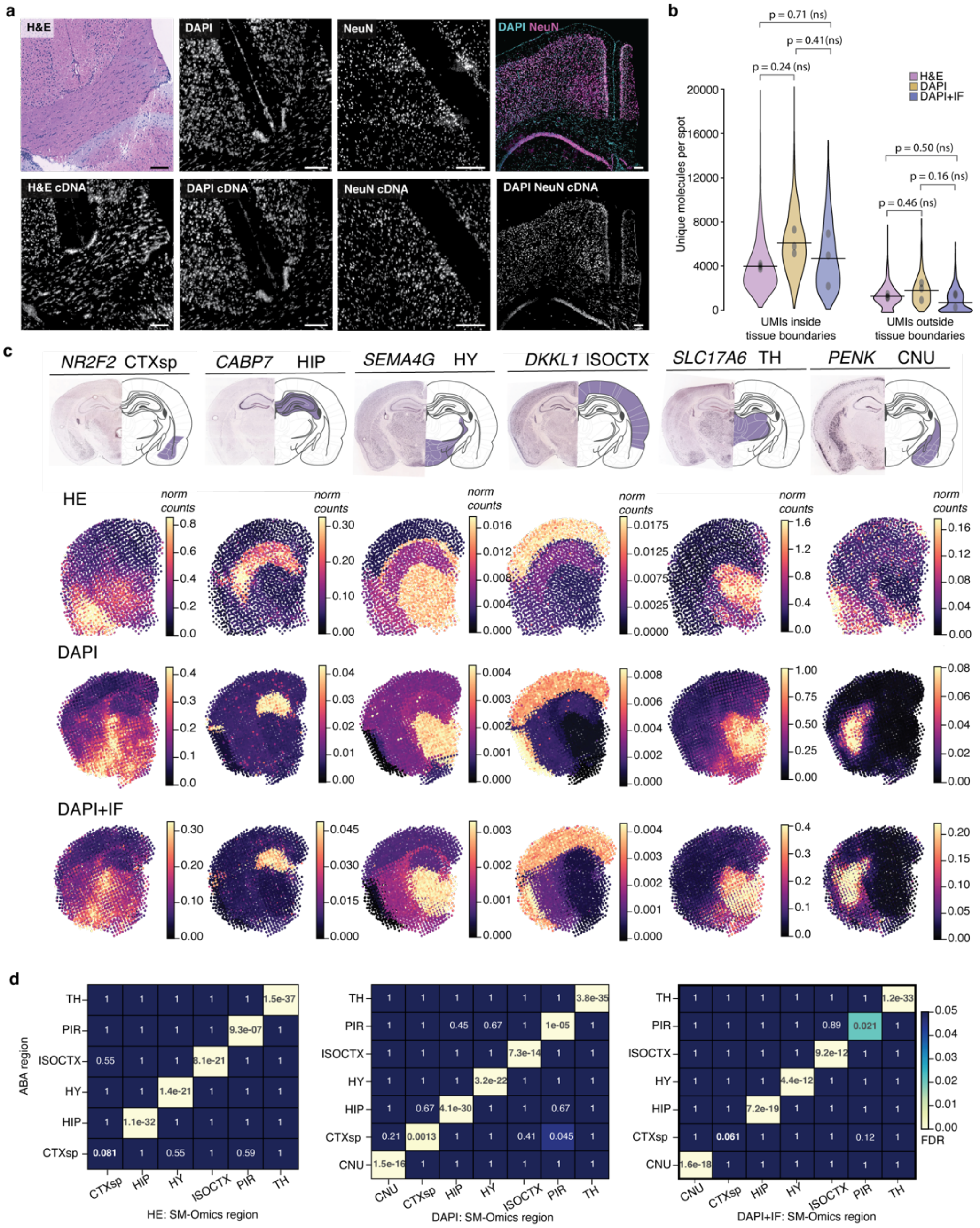

Supplementary Fig. 9. Feasibility and quality of combined antibody immunofluorescence and spatial transcriptomics measurements. (a) Feasibility of combined antibody IF and spatial transcriptomics *in situ* measurements. Top panel represents cortex region images in the following order (left to right): H&E stained, only DAPI stained, only NeuN stained and DAPI/NeuN stained tissues. Bottom panel shows fluorescent gene activity as Cy3 cDNA footprints corresponding to top panels**.** No significant differences were observed in Cy3 cDNA signal intensities between the staining conditions (n=3, Wilcoxon’s rank-sum test, p-value>0.05). Scale bars; 200µm. **(b)**

Comparison of number of unique molecules per spatial spot. Number of expressed unique molecules detected per spatial spot under and outside of the tissue boundaries for all spatial measurements in SM-Omics staining conditions: H&E (purple, n=3), DAPI (yellow, n=3) and a combined DAPI and IF stain (blue, n=3). Black dots represent summarized mean values for replicate libraries at the highest available sequencing saturation point. Horizontal line denotes the mean of the summarized library values (n=3) per condition. Statistical significance (Wald's test) markings are displayed: 0.05<p-value≤1 (ns). (**c**) Spatial gene expression patterns. *in situ* hybridization images from ABA (top) for DE genes in each morphological region (columns) followed by examples of SM-Omics spatial gene expression patterns (normalized expression shown in color scale) for the same DE genes detected and shown in ABA (Image credit: Allen Institute for Brain Science, **Methods**). Shown in rows are spatial gene expression patterns resulting for three different staining conditions as in **(b)**. **(d)** Gene expression specificity. Morphological gene expression signatures agree between SM-Omics and ABA for the all major layers and in all three staining conditions. Significance (FDR, Fisher's exact test, one-sided, color scale) for the overlap of genes associated with each layer in SM-Omics' three staining conditions: H&E (left), DAPI (middle) and DAPI+IF (right)(columns) and in ABA (rows). Annotated region abbreviations: CTXsp (cortical subplate), HIP (hippocampal formation), HY (hypothalamus), TH (thalamus), CNU (cerebral nuclei), ISOCTX (isocortex) and PIR (piriform area).

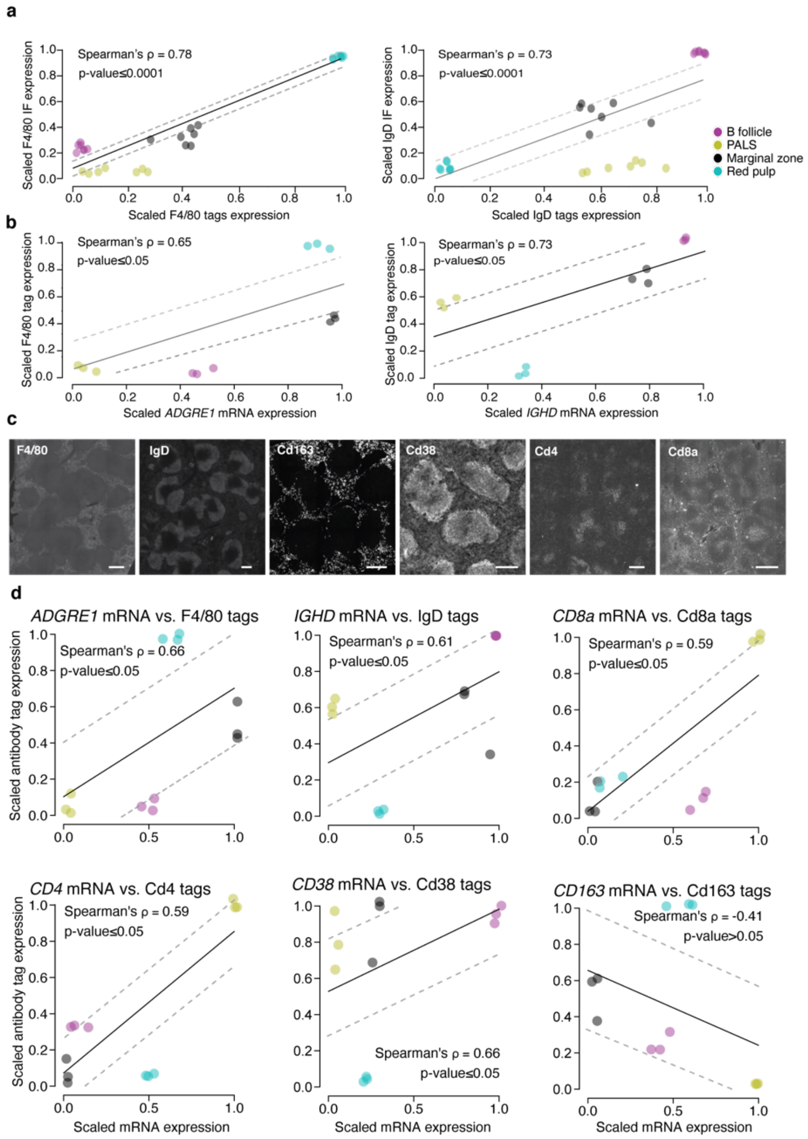

Supplementary Fig. 10. Performance of combined antibody immunofluorescence, DNA-barcoded antibodies and spatial transcriptomics measurements. (a) Antibody-based protein expression correlation. DNA-barcoded antibody (x axis, scaled normalized expression) and respective IF expression (y axis, scaled normalized expression) levels (mean scaled signal of all measurements) per tissue section (n=7, Methods) of each of two targets, F4/80 (left) and IgD (right) in each of four regions (spot color) in SM-Omics. **(b)** Two-plex gene and DNA-barcoded antibody signal correlation. Gene (x axis, scaled normalized expression) and respective DNA-barcoded antibody expression (y axis, scaled normalized expression) levels (mean scaled signal of all measurements) per tissue section (n=3, **Methods**) of each of two target combinations, F4/80/*ADGRE1* (left) and IgD/*IGHD* (right) in each of four regions (spot color, color code is shared with **(a)**) in a two-plex SM-Omics reaction. **(c)** IF images of six antibody clones staining adult spleen sections (n=3): F4/80, IgD, Cd163, Cd38, Cd4 and Cd8a. Scale bars denote 500µm. (**d**) Multiplex gene and DNA-barcoded antibody signal correlation. Gene (x axis, scaled normalized expression) and respective DNA-barcoded antibody expression (y axis, scaled normalized expression) levels (mean scaled signal of all measurements) per tissue section (n=3,

Methods) of each of six target combinations in a 6-plex SM-Omics reaction in each of four regions (spot color, color code is shared with **(a)**). Black line in (**a-b, d**) represents the linear regression line with respective standard deviations (gray lines).
