# Supplementary material for: SM-Omics is an automated platform for high-throughput spatial multi-omics
Source: Nat Commun. 2022 Feb 10;13:795. doi: 10.1038/s41467-022-28445-y (PMC8831571; doi:10.1038/s41467-022-28445-y)
Supplement: Supplementary file 2 — Reporting Summary [file 41467_2022_28445_MOESM2_ESM.pdf]

nature research

Corresponding author(s): Sanja Vickovic and Aviv Regev

Last updated by author(s): Jan 15, 2022

Reporting Summary

Nature Research wishes to improve the reproducibility of the work that we publish. This form provides structure for consistency and transparency in reporting. For further information on Nature Research policies, see our Editorial Policies and the Editorial Policy Checklist.

Statistics

For all statistical analyses, confirm that the following items are present in the figure legend, table legend, main text, or Methods section.

n/a	Confirmed	
☐	☒	The exact sample size (*n*) for each experimental group/condition, given as a discrete number and unit of measurement
☐	☒	A statement on whether measurements were taken from distinct samples or whether the same sample was measured repeatedly
☐	☒	The statistical test(s) used AND whether they are one- or two-sided<br>*Only common tests should be described solely by name; describe more complex techniques in the Methods section.*
☐	☒	A description of all covariates tested
☐	☒	A description of any assumptions or corrections, such as tests of normality and adjustment for multiple comparisons
☐	☒	A full description of the statistical parameters including central tendency (e.g. means) or other basic estimates (e.g. regression coefficient) AND variation (e.g. standard deviation) or associated estimates of uncertainty (e.g. confidence intervals)
☐	☒	For null hypothesis testing, the test statistic (e.g. *F*, *t*, *r*) with confidence intervals, effect sizes, degrees of freedom and *P* value noted<br>*Give P values as exact values whenever suitable.*
☐	☒	For Bayesian analysis, information on the choice of priors and Markov chain Monte Carlo settings
☐	☒	For hierarchical and complex designs, identification of the appropriate level for tests and full reporting of outcomes
☒	☐	Estimates of effect sizes (e.g. Cohen's *d*, Pearson's *r*), indicating how they were calculated

Our web collection on statistics for biologists contains articles on many of the points above.

Software and code

Policy information about availability of computer code

Data collection	Standard Illumina processing pipelines were used to collect pair-end sequencing data on a Nextseq 550 instrument. The BCL files from the sequencer were deposited in a NAS server where they were encrypted and sent to a backup server. The BCL files were then sent to a demultiplexing server where they were demultiplexed with bcl2fastq v2.17.1.14. The demultiplexed files were then sent to an analysis server where they merged by index prior analysis with the ST Pipeline (v1.7.6). Immunofluorescence and bright field images were collected Images of stained tissue sections on the SM-Omics slides were taken using a Metafer Vslide scanning system (MetaSystems, Germany) installed on an Axio Imager Z2 microscope (Carl Zeiss, Germany) using an LED transmitted light source and a CCD camera (BF scanning). All images were taken with the A-P 10x/0.25 Ph1 objective lens (Carl Zeiss, Germany). For fluorescent scanning, a PhotoFLuor LM-75 lightsource (89North, USA) was used in combination with a Plan-APOCHROMAT 20x/0.8 objective (Carl Zeiss, Germany). A configuration program was made to enable automatic tissue detection, focusing and scanning on all ST arrays present on a glass slide. In short, tissue detection was based on contrast as compared to normalized background in RGB channels. Upon finding maximum contrast in a 12-step spiral-like search window field of view (FOV) pattern, the automated focal alignment in every second of each FOV (4096x3000 px) was initiated. The alignment search considered the maximum contrast z-position as in-focus using 5µm stage intervals (n=19 focal planes). The BF scanning of the predefined ST array areas was done in a total of 48 FOVs and ~30sec in 3 channels (RGB); or fluorescent scanning of 228 FOVs and ~6min for 3 fluorescent channels. Images were stitched using 60µm overlap and linear blending between FOVs with the VSlide software (v1.0.0) and then extracted using jpg compression. Multiple ST slides can be processed in the same manner without any user input for a total of 6min processing time per H&E stained slide (3 channels) or 45min for fluorescently stained slide (3 channels), including image stitching.
Data analysis	ST, SM-Omics, Visium or antibody tag fastq reads were generated with bcl2fastq2. ST Pipeline v.1.7.6 was used to demultiplex the spatial barcodes and collapse duplicate UMI sequences for ST, SM-Omics and Visium. In short, 5nt trimmed R2 was used for mapping to the mouse genome (GRCm38 primary assembly available at https://www.ncbi.nlm.nih.gov/assembly/GCF_000001635.20/) using STAR (v2.6.0). After that, mapped reads were annotated using HTseq-count (v0.11.4) using the m11 gtf file (https://www.gencodegenes.org/mouse/release_M11.html). To collapse UMIs, the annotated reads needed to first be connected to a spatial barcode using a TagGD (v0.3.6) demultiplexer (k-mer 6, mismatches 2). Then, UMIs mapping to the same transcript and spatial barcode were collapsed using naive clustering with one mismatch allowed in the mapping process. The output file was a genes-by-barcode matrix that was used in all further processing

steps. To map antibody tags to their respective spatial barcodes, we used the tag quantification pipeline originally developed for CITE-Seq (v.1.4.3) available at https://github.com/Hoohm/CITE-seq-Count. The pipeline was run with default parameters (maximum Hamming distance of 1). We additionally provided the spatial barcodes and corrected the spatial mapping (1 mismatch) for a total of 1007 different barcodes. For efficient automated image registration and processing of spatial arrays, HE images were scaled to approximately 500x500 pixels using the imagemagick (https://imagemagick.org/index.php) mogrify command. Other image operations as mentioned below were performed using the R package imager (http://dahtah.github.io/imager/) unless specified differently. Scikit-image was used to process the H&E and respective fluorescent gene expression image and Ilastik (v1.3.3) used for quantitating immunofluorescence per SM-Omics spot. For comparisons between different protocols, raw data were first processed and downsampled and each replicate (at least n=3) from each condition (i.e. spatial RNA-seq protocol version) was represented by the counts mean at each of 9 different saturation points. Following processing, summarized counts data in each comparison were first scaled [0,1] and then used to estimate a generalized linear mixed model (glmm). We used a glmm (R package glmmTMB v1.1.1) modeled as a proportional binomial logit response between counts, protocol version (fixed effect) and replicate ID (random effect). Log proportions of annotated reads were used as offsets in the model. All glmm estimates were performed using the R stats package (v4.0.1) and Wald's p-values reported.

For manuscripts utilizing custom algorithms or software that are central to the research but not yet described in published literature, software must be made available to editors and reviewers. We strongly encourage code deposition in a community repository (e.g. GitHub). See the Nature Research guidelines for submitting code & software for further information.

Data

Policy information about availability of data

All manuscripts must include a data availability statement. This statement should provide the following information, where applicable:

- Accession codes, unique identifiers, or web links for publicly available datasets
- A list of figures that have associated raw data
- A description of any restrictions on data availability

Raw sequencing data is available at NCBI's Sequence Read Archive under accession PRJNA797464 [https://www.ncbi.nlm.nih.gov/sra/PRJNA797464]. All processed and source data generated in this study have been deposited in the Single Cell Portal under accession code SCP979 [https://portals.broadinstitute.org/single_cell/study/SCP979]. All other relevant data supporting the key findings of this study are available within the article and its Supplementary Information files.

Field-specific reporting

Please select the one below that is the best fit for your research. If you are not sure, read the appropriate sections before making your selection.

☒ Life sciences ☐ Behavioural & social sciences ☐ Ecological, evolutionary & environmental sciences

For a reference copy of the document with all sections, see nature.com/documents/nr-reporting-summary-flat.pdf

Life sciences study design

All studies must disclose on these points even when the disclosure is negative.

Sample size	At least three replicate experiments were used in the analysis using adult mice. In previous experimental designs and studies, we observed minimal variability for the obtained phenotypes among different samples on individually performed experiments using individual slides which we reasoned n=3 ("n" indicates distinct samples) will be sufficient to determine heuristics between the different methods (Lord et al, J Cell Biol (2020) 219 (6): e202001064)
Data exclusions	No data was excluded from the study.
Replication	We performed experiments with individual technical replicates ie. tissue sections (n=18 for MOB, n = 12 for HE stained cortex, n = 6 for NeuN stained cortex, n = 9 for DAPI stained cortex, n=7 for spleen). All findings could be replicated in this study.
Randomization	At least three samples were at randomly selected for each group in this study, with each biological replicate processed at least three times for experiments providing data on comparisons between the protocols.
Blinding	Analyses were not blinded, because the same researcher performed the experiment and analyzed the data. The collected measurements were quantifiable in nature, minimizing the chance of bias.

Reporting for specific materials, systems and methods

We require information from authors about some types of materials, experimental systems and methods used in many studies. Here, indicate whether each material, system or method listed is relevant to your study. If you are not sure if a list item applies to your research, read the appropriate section before selecting a response.

Materials & experimental systems

n/a	Involved in the study	
☐	☒	Antibodies
☒	☐	Eukaryotic cell lines
☒	☐	Palaeontology and archaeology
☐	☒	Animals and other organisms
☒	☐	Human research participants
☒	☐	Clinical data
☒	☐	Dual use research of concern

Methods

n/a	Involved in the study	
☒	☐	ChIP-seq
☒	☐	Flow cytometry
☒	☐	MRI-based neuroimaging

Antibodies

Antibodies used

NeuN, Clone EPR12763, Provider Abcam, ab177487 Alexa Fluor 647, Conc. 0.8 mg/ml, Dilution used 1:100
Nestin, Clone Rat-401, Provider Biolegend 655107 Alexa Fluor 647, Conc. 0.5 mg/ml Dilution used 1:100
F4/80, Clone BM8, Provider Biolegend 123117 APC, Conc. 0.2 mg/ml, Dilution used 1:100
IgD, Clone 11-26c.2a, Provider Biolegend 405707 Alexa Fluor 647, Conc. 0.5 mg/mL, Dilution used 1:100
CD4, Clone RM4-5, Provider Biolegend 100515 APC, Conc. 0.2 mg/ml, Dilution used 1:100
CD8a, Clone 53-6.7, Provider Biolegend 100711 APC, Conc. 0.2 mg/ml, Dilution used 1:100
Cd163, Clone S15049I, Provider Biolegend 142407 APC, Conc. 0.5 mg/mL, Dilution used 1:100
Cd38, Clone 90, Provider Biolegend 102711 APC, Conc. 0.2 mg/ml, Dilution used 1:100
F4/80, Clone BM8, Provider Biolegend 123153 TTAACTTCAGCCCGT, Conc. 0.5 mg/ml, Dilution used 1:100
IgD, Clone 11-26c.2a, Provider Biolegend 405745 TCATATCCGTTGTCC, Conc. 0.5 mg/ml, Dilution used 1:100
CD4, Clone RM4-5, Provider Biolegend 100569 AACAAGACCCTTGAG, Conc. 0.5 mg/ml, Dilution used 1:100
CD8a, Clone 53-6.7, Provider Biolegend 100773 TACCCGTAATAGCGT, Conc. 0.5 mg/ml, Dilution used 1:100
Cd163, Clone S15049I, Provider Biolegend 155303 GAGCAAGATTAAGAC, Conc. 0.5 mg/ml, Dilution used 1:100
Cd38, Clone 90, Provider Biolegend 102733 CGTATCCGTCTCCTA, Conc. 0.5 mg/ml, Dilution used 1:100

Validation

NeuN, Clone EPR12763, Provider Abcam, Supplier verified reactivity with mouse, Suitable for: Flow Cyt (Intra), IHC (PFA fixed), mIHC, IHC-P, WB, ICC/IF, IHC-Fr
Nestin, Clone Rat-401, Provider Biolegend, Supplier verified reactivity with mouse, Suitable for: IHC-P
F4/80, Clone BM8, Provider Biolegend, Supplier verified reactivity with mouse, Suitable for: FC
IgD, Clone 11-26c.2a, Provider Biolegend, Supplier verified reactivity with mouse, Suitable for: FC and 3D IHC
CD4, Clone RM4-5, Provider Biolegend , Supplier verified reactivity with mouse, Suitable for: FC
CD8a, Clone 53-6.7, Provider Biolegend, Supplier verified reactivity with mouse, Suitable for: FC
Cd163, Clone S15049I, Provider Biolegend, Supplier verified reactivity with mouse Suitable for: FC, IHC-F and 3D IHC
Cd38, Clone 90, Provider Biolegend , Supplier verified reactivity with mouse, Suitable for: FC
F4/80, Clone BM8, Provider Biolegend, Supplier verified reactivity with mouse, Suitable for: PG
IgD, Clone 11-26c.2a, Provider Biolegend, Supplier verified reactivity with mouse, Suitable for: PG
CD4, Clone RM4-5, Provider Biolegend , Supplier verified reactivity with mouse, Suitable for: PG
CD8a, Clone 53-6.7, Provider Biolegend, Supplier verified reactivity with mouse, Suitable for: PG
Cd163, Clone S15049I, Provider Biolegend, Supplier verified reactivity with mouse, Suitable for: PG
Cd38, Clone 90, Provider Biolegend, Supplier verified reactivity with mouse, Suitable for: PG

Animals and other organisms

Policy information about studies involving animals; ARRIVE guidelines recommended for reporting animal research

Laboratory animals

Adult C57BL/6J mice were used in all experiments. Details about experiment performed, sample ID, mouse ID , sex and age can be found as detailed bellow:
10015CN89_C2 Spatial polyT RNA-Seq of Mus musculus: p84 adult brain cortex from male individual animal ID CortexM2
10015CN89_D2 Spatial polyT RNA-Seq of Mus musculus: p84 adult brain cortex from male individual animal ID CortexM2
10015CN89_E2 Spatial polyT RNA-Seq of Mus musculus: p84 adult brain cortex from male individual animal ID CortexM2
10015CN108fl_C1 Spatial polyT RNA-Seq of Mus musculus: p56 adult brain cortex from male individual animal ID M18
10015CN108fl_C2 Spatial polyT RNA-Seq of Mus musculus: p56 adult brain cortex from male individual animal ID M18
10015CN108fl_D1 Spatial polyT RNA-Seq of Mus musculus: p56 adult brain cortex from male individual animal ID M18
10015CN108fl_D2 Spatial polyT RNA-Seq of Mus musculus: p56 adult brain cortex from male individual animal ID M18
10015CN108fl_E1 Spatial polyT RNA-Seq of Mus musculus: p56 adult brain cortex from male individual animal ID M18
10015CN108fl_E2 Spatial polyT RNA-Seq of Mus musculus: p56 adult brain cortex from male individual animal ID M18
10015CN78_C1 Spatial polyT RNA-Seq of Mus musculus: p84 adult brain cortex from male individual animal ID CortexM2
10015CN78_C2 Spatial polyT RNA-Seq of Mus musculus: p84 adult brain cortex from male individual animal ID CortexM2
10015CN78_D1 Spatial polyT RNA-Seq of Mus musculus: p84 adult brain cortex from male individual animal ID CortexM2
10015CN78_D2 Spatial polyT RNA-Seq of Mus musculus: p84 adult brain cortex from male individual animal ID CortexM2
10015CN78_E1 Spatial polyT RNA-Seq of Mus musculus: p84 adult brain cortex from male individual animal ID CortexM2
10015CN78_E2 Spatial polyT RNA-Seq of Mus musculus: p84 adult brain cortex from male individual animal ID CortexM2
10015CN108_C1 Spatial polyT RNA-Seq of Mus musculus: p56 adult brain cortex from male individual animal ID M18

	10015CN108_C2 Spatial polyT RNA-Seq of Mus musculus: p56 adult brain cortex from male individual animal ID M18<br>10015CN108_D1 Spatial polyT RNA-Seq of Mus musculus: p56 adult brain cortex from male individual animal ID M18<br>10015CN108_D2 Spatial polyT RNA-Seq of Mus musculus: p56 adult brain cortex from male individual animal ID M18<br>10015CN108_E1 Spatial polyT RNA-Seq of Mus musculus: p56 adult brain cortex from male individual animal ID M18<br>10015CN108_E2 Spatial polyT RNA-Seq of Mus musculus: p56 adult brain cortex from male individual animal ID M18<br>10015CN89_C1 Spatial polyT RNA-Seq of Mus musculus: p84 adult brain cortex from male individual animal ID CortexM2<br>10015CN89_D1 Spatial polyT RNA-Seq of Mus musculus: p84 adult brain cortex from male individual animal ID CortexM2<br>10015CN89_E1 Spatial polyT RNA-Seq of Mus musculus: p84 adult brain cortex from male individual animal ID CortexM2<br>10015CN90_C1 Spatial polyT RNA-Seq of Mus musculus: p84 adult brain cortex from male individual animal ID CortexM2<br>10015CN90_C2 Spatial polyT RNA-Seq of Mus musculus: p84 adult brain cortex from male individual animal ID CortexM2<br>10015CN90_D1 Spatial polyT RNA-Seq of Mus musculus: p84 adult brain cortex from male individual animal ID CortexM2<br>10015CN90_D2 Spatial polyT RNA-Seq of Mus musculus: p84 adult brain cortex from male individual animal ID CortexM2<br>10015CN90_E1 Spatial polyT RNA-Seq of Mus musculus: p84 adult brain cortex from male individual animal ID CortexM2<br>10015CN90_E2 Spatial polyT RNA-Seq of Mus musculus: p84 adult brain cortex from male individual animal ID CortexM2<br>10015CN83_C1 Spatial polyT RNA-Seq of Mus musculus: p56 adult brain MOB from female individual animal ID M9<br>10015CN83_C2 Spatial polyT RNA-Seq of Mus musculus: p56 adult brain MOB from female individual animal ID M9<br>10015CN83_D1 Spatial polyT RNA-Seq of Mus musculus: p56 adult brain MOB from female individual animal ID M9<br>10015CN83_D2 Spatial polyT RNA-Seq of Mus musculus: p56 adult brain MOB from female individual animal ID M9<br>10015CN83_E1 Spatial polyT RNA-Seq of Mus musculus: p56 adult brain MOB from female individual animal ID M9<br>10015CN83_E2 Spatial polyT RNA-Seq of Mus musculus: p56 adult brain MOB from female individual animal ID M9<br>10015CN84_C1 Spatial polyT RNA-Seq of Mus musculus: p56 adult brain MOB from female individual animal ID M9<br>10015CN84_C2 Spatial polyT RNA-Seq of Mus musculus: p56 adult brain MOB from female individual animal ID M9<br>10015CN84_D1 Spatial polyT RNA-Seq of Mus musculus: p56 adult brain MOB from female individual animal ID M9<br>10015CN84_D2 Spatial polyT RNA-Seq of Mus musculus: p56 adult brain MOB from female individual animal ID M9<br>10015CN84_E2 Spatial polyT RNA-Seq of Mus musculus: p56 adult brain MOB from female individual animal ID M9<br>10015CN85_C1 Spatial polyT RNA-Seq of Mus musculus: p56 adult brain MOB from female individual animal ID M9<br>10015CN85_C2 Spatial polyT RNA-Seq of Mus musculus: p56 adult brain MOB from female individual animal ID M9<br>10015CN85_D1 Spatial polyT RNA-Seq of Mus musculus: p56 adult brain MOB from female individual animal ID M9<br>10015CN85_E1 Spatial polyT RNA-Seq of Mus musculus: p56 adult brain MOB from female individual animal ID M9<br>10015CN85_E2 Spatial polyT RNA-Seq of Mus musculus: p56 adult brain MOB from female individual animal ID M9<br>10015CN60_C2 Spatial polyT RNA-Seq of Mus musculus: p56 adult brain MOB from female individual animal ID M10<br>10015CN60_E2 Spatial polyT RNA-Seq of Mus musculus: p56 adult brain MOB from female individual animal ID M10<br>V19S23-065A1 Spatial polyT RNA-Seq of Mus musculus: p84 adult brain cortex from male individual animal ID CortexM2<br>V19S23-065B1 Spatial polyT RNA-Seq of Mus musculus: p84 adult brain cortex from male individual animal ID CortexM2<br>V19S23-065D1 Spatial polyT RNA-Seq of Mus musculus: p84 adult brain cortex from male individual animal ID CortexM2<br>10005CN48_C1 Spatial polyT RNA-Seq of Mus musculus: p84 adult brain MOB from female individual animal ID M4<br>10005CN48_D1 Spatial polyT RNA-Seq of Mus musculus: p84 adult brain MOB from female individual animal ID M4<br>10005CN48_E1 Spatial polyT RNA-Seq of Mus musculus: p84 adult brain MOB from female individual animal ID M4<br>5005CN107_D2 Spatial polyT RNA-Seq of Mus musculus: p84 adult brain cortex from male individual animal ID CortexM2<br>10015CN87_C1 Spatial polyT RNA-Seq of Mus musculus: p35 adult spleen from male individual animal ID Spleen<br>10015CN87_D1 Spatial polyT RNA-Seq of Mus musculus: p35 adult spleen from male individual animal ID Spleen<br>10015CN87_D2 Spatial polyT RNA-Seq of Mus musculus: p35 adult spleen from male individual animal ID Spleen<br>10015CN87_E2 Spatial polyT RNA-Seq of Mus musculus: p35 adult spleen from male individual animal ID Spleen<br>10015CN87_C1 Spatial polyT Antibody-tag of Mus musculus: p35 adult spleen from male individual animal ID Spleen<br>10015CN87_D1 Spatial polyT Antibody-tag of Mus musculus: p35 adult spleen from male individual animal ID Spleen<br>10015CN87_D2 Spatial polyT Antibody-tag of Mus musculus: p35 adult spleen from male individual animal ID Spleen<br>10015CN87_E2 Spatial polyT Antibody-tag of Mus musculus: p35 adult spleen from male individual animal ID Spleen<br>10015CN77_C1 Spatial polyT Antibody-tag of Mus musculus: p35 adult spleen from male individual animal ID Spleen<br>10015CN77_D1 Spatial polyT Antibody-tag of Mus musculus: p35 adult spleen from male individual animal ID Spleen<br>10015CN77_E1 Spatial polyT Antibody-tag of Mus musculus: p35 adult spleen from male individual animal ID Spleen<br>10015CN88_D1 Spatial polyT RNA-Seq of Mus musculus: p35 adult spleen from male individual animal ID Spleen<br>10015CN88_D2 Spatial polyT RNA-Seq of Mus musculus: p35 adult spleen from male individual animal ID Spleen<br>10015CN88_E1 Spatial polyT RNA-Seq of Mus musculus: p35 adult spleen from male individual animal ID Spleen<br>10015CN88_D1 Spatial polyT Antibody-tag of Mus musculus: p35 adult spleen from male individual animal ID Spleen<br>10015CN88_D2 Spatial polyT Antibody-tag of Mus musculus: p35 adult spleen from male individual animal ID Spleen<br>10015CN88_E1 Spatial polyT Antibody-tag of Mus musculus: p35 adult spleen from male individual animal ID Spleen<br>10015CN45_C1 Spatial polyT RNA-Seq of Mus musculus: p84 adult colorectal cancer from male individual animal ID Colon<br>10015CN45_D1 Spatial polyT RNA-Seq of Mus musculus: p84 adult colorectal cancer from male individual animal ID Colon<br>10015CN45_E1 Spatial polyT RNA-Seq of Mus musculus: p84 adult colorectal cancer from male individual animal ID Colon<br>10015CN45_C2 Spatial polyT RNA-Seq of Mus musculus: p84 adult colorectal cancer from male individual animal ID Colon<br>10015CN45_D2 Spatial polyT RNA-Seq of Mus musculus: p84 adult colorectal cancer from male individual animal ID Colon<br>10015CN45_E2 Spatial polyT RNA-Seq of Mus musculus: p84 adult colorectal cancer from male individual animal ID Colon
Wild animals	The study did not involve wild animals.
Field-collected samples	The study did not involve samples collected from the field.
Ethics oversight	All work involving C57BL/6J mice was performed under specific-pathogen-free conditions and the guidelines of the Division of Comparative Medicine, in accordance with the Institutional Animal Care and Use Committees (IACUC) relevant guidelines at the

Broad Institute of Harvard and MIT, and consistent with the Guide for Care and Use of Laboratory Animals, National Research Council, 1996 (institutional animal welfare assurance no. A4711-01), with protocol 0122-10-16.

Note that full information on the approval of the study protocol must also be provided in the manuscript.
