# Supplementary material for: SM-Omics is an automated platform for high-throughput spatial multi-omics
Source: Nat Commun. 2022 Feb 10;13:795. doi: 10.1038/s41467-022-28445-y (PMC8831571; doi:10.1038/s41467-022-28445-y)
Supplement: Supplementary file 3 — Description of Additional Supplementary Files [file 41467_2022_28445_MOESM3_ESM.pdf]

Description of Additional Supplementary Files

File Name: Supplementary Data 1
Description: Data quality metrics, processing time, throughput and cost for different spatial technologies.

File Name: Supplementary Data 2
Description: UMI and IF counts per spatial region.

File Name: Supplementary Data 3
Description: List of reagents and antibody staining conditions.
